# Pyrethroid resistance in *Aedes aegypti*: genetic mechanisms worldwide, and recommendations for effective vector control

**DOI:** 10.1186/s13071-025-07010-8

**Published:** 2025-10-14

**Authors:** Jonathan Rene Hernandez, Patricia Victoria Pietrantonio

**Affiliations:** Department of Entomology, Texas A&M University, College Station, TX 77843-2475 USA

**Keywords:** *Aedes aegypti*, Pyrethroid resistance, *kdr* mutations, Voltage-gated sodium channel, Insecticide resistance mechanisms, Mosquito control strategies, Insecticide bioassays, Resistance ratios, Integrated vector management

## Abstract

**Background:**

The* Aedes aegypti* mosquito, a primary vector of arboviruses such as dengue, Zika, yellow fever, and chikungunya, poses a significant public health threat worldwide. Its adaptability and genetic diversity complicate control efforts, enabling rapid resistance evolution. Pyrethroid insecticides, a cornerstone of vector control, target voltage-gated sodium channels, yet resistance driven by knockdown resistance (*kdr*) mutations and detoxification mechanisms have undermined their efficacy.

**Methods:**

This review summarizes findings from a targeted literature search, exploring the genetic and molecular mechanisms driving pyrethroid resistance worldwide, focusing on* kdr* mutations.

**Results:**

Over twenty distinct* kdr* alleles were reported across global populations, including those functionally confirmed on the recombinant sodium channel such as V253F, V410L, L982W, I1011M, V1016G and F1534C. Indicators of the global impact of pyrethroid resistance include the field selection of highly resistant populations in which* kdr* mutation frequencies exceed 90%, deltamethrin resistance ratios as high as 249-fold, and permethrin resistance exceeding 500-fold. In laboratory-selected pyrethroid-resistant strains, resistance ratios can surpass 1,000-fold.

**Conclusions:**

We provide an updated status of pyrethroid resistance in* Ae. aegypti* and a framework on how the results of molecular tests and toxicity bioassays can be applied to practical mosquito control programs. Control strategies must integrate multidisciplinary approaches, including Integrated Vector Management (IVM), which emphasizes targeted interventions, community engagement, and sustainable practices. Despite advances in analyzing resistance, very few studies measure frequency of genotypes, determine phenotypic resistance (resistance ratios), and assess standardized field efficacy in the same populations, including field measurements of pesticide deposited, leaving a critical implementation gap. This lack of integration creates major gaps in translating laboratory resistance diagnostics into actionable field control decisions. Empirical data on how cuticular thickening and behavioral avoidance alter post-treatment survivorship are especially sparse, limiting the predictive power of current methodologies. By assessing the current understanding of pyrethroid resistance in* Ae. aegypti,* this review informs the development of resilient, evidence-based interventions to mitigate the public health impact of diseases transmitted by* Ae. aegypti.*

**Graphical Abstract:**

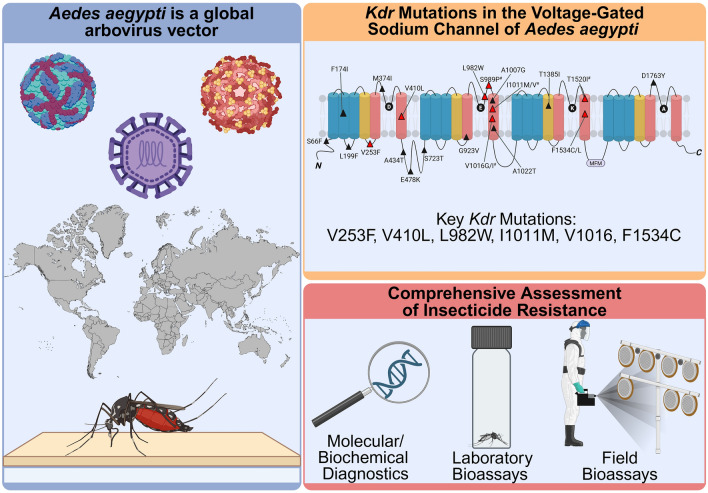

## Background

### *Aedes aegypti*, the yellow fever mosquito

The yellow fever mosquito, *Aedes aegypti,* is a primary vector of emerging arboviruses that threaten to infect millions of people worldwide. Half of the world’s population in more than 125 countries is at risk of arboviral transmission from *Ae. aegypti* [1–3]. This species thrives in warm, humid climates, particularly during summer months that are optimal for larval habitats [4]. *Aedes aegypti* is well-adapted to urban environments and preferentially lays eggs in man-made containers, including tires, buckets, drains and water tanks [5–7]. While the blood-feeding females prefer feeding on humans, they can feed opportunistically on other vertebrates [8–11].

*Aedes aegypti* has two major subspecies: the ancestral *Aedes aegypti formosus* (*Aaf*) found in sub-Saharan Africa and *Aedes aegypti aegypti* (*Aaa*), which has expanded beyond Africa. *Aaf* is sylvatic and prefers blood-feeding on non-human vertebrates [12]. Although *Aaa* originates from the forests of sub-Saharan Africa, it has become widespread across the tropics and sub-tropics owing to trade, travel, and urbanization [13–16]. Molecular studies support that the migration of *Aaa* (from this point on referred to as *Ae. aegypti*) to the western hemisphere was from a single introduction event [13]. Genetically, *Ae. aegypti* outside of Africa are genetically sub-divided into three major groups: North America, South America, and Asia [13].

In the USA, the *Ae. aegypti* mosquito is predominantly distributed across the southeastern states, including Florida, Georgia, Alabama, Mississippi, Louisiana, and Texas, with a range extending along the Atlantic coastal plain as far north as Maryland and in parts of the Southwest, specifically Arizona and southern California [17]. The earliest colonization event for *Ae. aegypti* was likely in Florida, due to ship trade and gene flow from the Caribbean region and South America, followed by a series of westward invasions that served as founder events [18]. North American populations can be genetically categorized into five regional groups which are: the Southeast (Louisiana, Florida, and Georgia), Central (Texas and New Mexico), southwest (Arizona), Southern California (Nevada and Southern California), and northern California (Northern California) regions [18, 19]. The fact that California has two distinct populations suggests that *Ae. aegypti* had multiple successful introduction events [19].

Historical control efforts to reduce *Ae. aegypti* populations in the USA During the 1950s And 1960s included widespread DDT sprays [20, 21]. These efforts reduced the abundance and distribution of *Ae. aegypti*, but the species was not eliminated. By the 1970s, *Ae. aegypti* were able to re-infest most American counties due to insecticide-resistant survivors and migration from untreated areas. While insecticide resistance was a factor, the mosquito’s success as an invader is also owing to biological traits that are perfectly suited to human-dominated landscapes, as mentioned earlier. These include its preference for laying eggs in small, often cryptic, man-made water containers and the ability of those eggs to survive drying out for months, ensuring both local persistence and easy dispersal through the movement of humans and transport of goods [22]. Effective control strategies must therefore address both genetic resistance mechanisms and the ecological and behavioral traits that allow *Ae. aegypti* to thrive in human environments, as its adaptability poses a significant public health threat [23–25].

### Genetic diversity and population structure of *Ae. aegypti*

Genetic diversity of *Ae. aegypti* has significant implications for its vector competence, or the ability of an arthropod vector to become infected with, replicate, and transmit a pathogen to vertebrates [26]. Variations in genes related to immune response, metabolism, and behavior can influence a mosquito’s susceptibility to infection and its capacity to transmit pathogens to humans [27]. Additionally, genetic diversity influences a population’s resistance to insecticides, which complicates control efforts [28]. Populations with high genetic variability are more likely to develop and sustain resistance to chemical interventions, making effective management difficult.

Understanding the genetic structure of different *Ae. aegypti* populations is essential for developing and efficiently implementing control measures within geographic limits necessary to disrupt transmission. The utilization of genetic markers, including allozymes, mtDNA, microsatellites, and single nucleotide polymorphisms (SNPs), has advanced our understanding of the genetic structure of *Ae. aegypti* [29, 30]. These markers can provide information on how genetic variability can influence pathogen transmission, gene flow, and insecticide resistance that might affect vector competence and vector control strategies [31, 32].

*Aedes aegypti* exhibits considerable genetic variation both within and among populations across its global range. In Africa, the mosquito’s native region, *Ae. aegypti* populations tend to display higher genetic diversity, particularly in genes associated with immune function and ecological adaptation, compared with those in other parts of the world owing to a longer evolutionary history and larger ancestral populations [33]. For instance, samples from Gabon, Kenya, and Senegal display significant genomic differentiation, and only limited admixture is observed among populations with differing levels of human host preference [34, 35]. Conversely, in regions outside of Africa where *Ae. aegypti* has been introduced more recently, genetic diversity tends to be lower, reflecting founder effects and bottlenecks associated with colonization events [18, 33, 36]. However, ongoing gene flow between populations, facilitated by human movement and trade, can introduce new genetic material, thereby maintaining or even increasing diversity. Additionally, in stable urban populations with a high abundance of humans, mosquito genetic diversity can expand, leading to greater heterogeneity within the population [37].

In more recently established areas, such as the USA, *Ae. aegypti* populations show strong genetic structure similarities among populations across different cities from Florida, despite long distances between cities [38]. This is due to major interstate highways facilitating movement, such as transporting used tires that contain desiccation-resistant eggs [39]. However, *Ae. aegypti* gene flow in Brazil is reported to be low among areas with and without dengue, despite heavy roads and railway connections [40]. Since *Ae. aegypti* populations do not disperse far from their emergence sites and possess a low effective population size [41], genetic differentiation at relatively fine scales may also be the result of low levels of gene flow among distinct populations. Similar patterns have been observed in other regions [42]. For instance, across Latin America, urban fragmentation and vector control efforts contribute to genetic isolation of mosquito populations, despite high human mobility [43]. Thus, throughout the world, gene flow may be more constrained by environmental barriers and uneven urban development, which complicate coordinated vector control [42, 44]. These issues challenge vector control, as strategies effective in one area may not necessarily be applicable with equivalent success to area-wide pest control efforts. This review provides the updated status of pyrethroid resistance in *Ae. aegypti* and a framework on how the results of molecular tests and toxicity bioassay can be applied to practical mosquito control programs.

## Methods

Targeted literature searches were conducted using keyword-based strategies across academic databases such as Google Scholar and PubMed.

For the detailed information on *kdr* (knockdown resistance) mutations, the following specific search terms were used: “*Aedes aegypti*,” “sodium channel,” and “*kdr*”. Articles were selected for their relevance to the genetic and molecular characterization of *kdr* mutations and their association with pyrethroid resistance in *Ae. aegypti*.

For the compilation of permethrin and deltamethrin resistance ratios in *Aedes aegypti*, a separate and focused search was conducted using the terms: “*Aedes aegypti*,” “resistance ratio,” “permethrin,” “deltamethrin,” and “pyrethroids”. For both the *kdr* mutation and pyrethroid resistance searches, Boolean operators were utilized for refinement, and no date restrictions were applied. Studies were selected based on their reporting of quantitative resistance metrics related to permethrin and deltamethrin susceptibility in *Ae. aegypti*.

### Pathogens transmitted by *Aedes aegypti*

*Aedes aegypti* (L.) is the primary vector of various arboviruses including dengue (DENV), yellow fever (YFV), chikungunya (CHIKV), and Zika (ZIKV) [45–48]. Dengue is endemic in over 110 countries with An estimated 50–528 million infections globally each year, leading to tens of thousands of deaths, primarily in tropical and subtropical regions across Asia, Latin America, and Africa [49, 50]. Recent outbreaks have seen significant surges in incidence in countries such as Brazil and India [51, 52]. Yellow fever, although largely preventable by vaccination, remains a public health challenge in regions with low vaccination coverage, particularly in parts of South America and in Sub-Saharan Africa, where sporadic outbreaks continue to cause significant mortality, Such as the Angola and Democratic Republic of Congo outbreaks of 2016 [53–56]. Chikungunya, characterized by severe and often chronic joint pain, is prevalent in Africa, Asia, and the Americas, with outbreaks occurring frequently in Latin America and the Caribbean [57–59]. Zika virus gained prominence During the 2015–2016 outbreak owing to its association with microcephaly and Guillain–Barré syndrome [60, 61]. While the initial epidemic peaked in the Americas, particularly Brazil, Colombia, and Puerto Rico, sustained low-level transmission continues in many affected areas, posing ongoing risks, especially for pregnant women [62, 63]. Comprehensive reviews on these diseases highlight their epidemiology, clinical manifestations, and the critical role of *Ae. aegypti* in their transmission [64–68].

*Aedes aegypti* is also a vector for other arboviral diseases, including Mayaro, Ross River, Barmah Forest, and Usutu viruses [27]. *Aedes aegypti* vector competency studies supported the role of transovarial transmission of La Crosse virus [69]. Outbreaks of these other arboviral diseases are generally less widespread and less severe than those of the major diseases previously mentioned [70].

### Challenges in controlling *Ae. aegypti* populations

Public health agencies play critical roles in controlling mosquito populations to prevent the spread of mosquito-borne diseases. Before the 1970s, traditional mosquito control approaches relied on chemical control interventions with synthetic pesticides [71]. Now, mosquito control agencies utilize a science-based multidisciplinary approach to control mosquitoes at all life stages, known as integrated vector management (IVM) [72]. However, insecticide use is not limited to public health interventions. Aside from targeted insecticide applications from public health agencies, mosquitoes can also be affected by pesticides used in agriculture [73, 74]. Furthermore, in many endemic regions, household and commercial use of insecticides, particularly aerosol sprays and repellents, contribute substantially to the overall selection pressure on mosquito populations [75]. Studies in Brazil have demonstrated that domestic insecticide use can drive the emergence and spread of resistance in *Ae. aegypti* populations even without formal vector control programs [76].

The relationship between pyrethroid resistance in *Ae. aegypti* and arboviral infection further complicates vector control. This relationship is context dependent, varying by resistance mechanism, virus type, and mosquito strain [77]. Several studies indicate that resistance, particularly metabolic detoxification and *kdr* mutations, can enhance *Ae. aegypti* susceptibility to viruses, such as dengue and Zika, increasing infection and dissemination rates in resistant mosquitoes compared with susceptible ones [78–80]. In contrast, target-site mutations, such as V1016I and F1534C, have also been associated with reduced dengue vector competence in some field populations, possibly due to physiological trade-offs or altered viral susceptibility [81, 82]. Other studies report no significant differences in arbovirus infection or dissemination between resistant and susceptible mosquitoes, including for Zika and yellow fever, suggesting that resistance does not universally alter vector competence [83, 84]. These conflicting findings underscore the need for additional mechanistic studies, particularly for viruses such as Chikungunya and Yellow Fever, where current evidence is limited, to better understand how specific resistance pathways shape arboviral transmission dynamics. Effectively managing mosquito populations, thereby requires integrated strategies that account for both viral epidemiology and the evolving challenge of insecticide resistance.

### Integrated vector management practices

IVM adapts principles from Integrated Pest Management (IPM) to mosquito control, combining biological, chemical, and environmental strategies to manage vector populations and reduce disease transmission [85, 86]. This approach can be applied under multiple scenarios with a variety of disease vectors [87]. A comprehensive IVM program includes inspection, identification, monitoring, action, and evaluation (Table 1) [86, 88].

**Table 1:** Framework for integrated vector management (IVM) program strategies

Step	Summary	Methods
1. Inspection	Inspection involves assessing risk factors for vector-borne diseases through site visits, mosquito specimen collection, and environmental evaluations. This step determines the need for vector control programs and prevents ineffective or environmentally damaging actions	Site visits Mosquito specimen collection Environmental assessments
2. Identification	Identification focuses on detecting vectors and diseases using molecular techniques (e.g., ELISAs, RT-PCR) and morphological analysis. GIS mapping and surveillance data help pinpoint high-risk areas, allowing targeted control strategies. During this step, the action thresholds are set	ELISAs RT-PCR Morphological analysis GIS mapping Surveillance data
3. Monitoring	Routine monitoring ensures early detection of vector populations and diseases. Monitoring can be done with methods utilized in the identification step. Various surveillance techniques, including traps, molecular diagnostics, and AI technologies are employed to track mosquito presence and pathogen transmission	Traps Molecular diagnostics AI technologies Field collections
4. Action	Action involves applying sustainable control methods, and prioritizing community engagement and non-chemical techniques such as source reduction and biological control. Genetic control strategies and chemical control (as a last resort) are also considered	Community engagement Source reduction Biological control Genetic strategies Chemical interventions
5. Evaluation	Evaluation measures the effectiveness of control interventions using pre- and post-treatment comparisons. Metrics used in evaluation include mosquito population changes, disease incidence reduction, cost-effectiveness, and insecticide resistance assessments. This is iterative and cycles back to the inspection step	Pre- and post-treatment data comparisons Disease incidence tracking Cost-effectiveness analysis Insecticide resistance assessments

Public health agencies conduct crucial activities in parallel with mosquito control, including mosquito surveillance, community outreach and stakeholder engagement [88]. Importantly, chemical control methods should be reserved as a last resort in IVM strategies to reduce mosquito populations only when necessary. In practice, the use of pesticides as a last resort can vary. Some applicators may prioritize nonchemical methods, while others might rely more heavily on pesticides owing to cost, convenience, or immediate effectiveness [89]. Larvicides applied to water sources kill mosquito larvae before they mature into adults, while adulticides target adult mosquitoes.

Chemical control of *Ae. aegypti* larvae includes larvicides that act on the nervous system, juvenile hormone analogues (*e.g.*, Methoprene), and chitin synthesis inhibitors [90, 91]. In addition to these chemical methods, another approach involves the use of microbial larvicides such as *Bacillus thuringiensis israelensis* (Bti) and *Bacillus sphaericus* [92, 93]. These bacteria produce protein toxins that, upon ingestion by mosquito larvae, specifically target and destroy the cells of the midgut, leading to rapid mortality. Collectively, these diverse methods are fundamental to many integrated mosquito management programs, providing crucial tools for targeting and controlling larval populations.

Some of the most popular methods of applying adulticides (typically neurotoxicants, e.g., pyrethroids and organophosphates) include thermal fogging, ultra-low volume (ULV) spraying, aerial spraying, and residual barrier (primarily used against *Anopheles* mosquitoes in malaria campaigns in Africa) [94]. Until recently, public health agencies in the USA have had access to only two classes of synthetic insecticides for adult mosquito control: organophosphates (IRAC Group 1B) and pyrethroids (IRAC Group 3A). However, the array of available adulticides recently expanded with the introduction of new products designed to address insecticide resistance.

The recently introduced abamectin (IRAC Group 6, glutamate-gated chloride channel allosteric modulators), for mosquito control is used in novel formulations [95, 96]. A recent example is ReMoa Tri, which combines fenpropathrin (a pyrethroid) with abamectin (a macrocyclic lactone) and a patented C8910 fatty acid blend [95]. While still incorporating a pyrethroid, the inclusion of abamectin introduces a distinct mode of action (IRAC Group 6), that of a glutamate-gated chloride channel allosteric modulator. The fatty acid blend further contributes by enhancing efficacy and potentially acting as a synergist. This multi-modal approach is critical for managing and combating widespread pyrethroid resistance in mosquito populations [95, 97].

Beyond pyrethroids, other chemical classes of adulticides are also employed against *Ae. aegypti*, particularly for resistance management or in specific operational contexts [98, 99]. These primarily include carbamates (e.g., propoxur, bendiocarb; IRAC Group 1A, acetylcholinesterase inhibitors) organophosphates (e.g., malathion, naled; IRAC Group 1B, acetylcholinesterase inhibitors), .Although these other modes of action are essential elements of integrated vector management, this review focuses specifically on pyrethroids. However, given widespread pyrethroid resistance discussed herein, organophosphates and abamectin remain critical control tools for *Ae. aegypti*.

Another chemical control strategy is the use of attractive toxic sugar baits (ATSBs) that use sugar, an attractant, and an oral toxicant to kill mosquitoes, which include males and females [100]. Similarly, lethal ovitraps attract gravid female mosquitoes to artificial breeding sites containing a lethal agent, effectively targeting egg-laying females and preventing the emergence of new adults [101, 102].

### Pyrethroids

Pyrethroids are the most frequently used class of insecticides to control *Ae. aegypti*. Pyrethroids comprise over 30% of the global insecticide use owing to their low toxicity to humans and high toxicity to insects [103, 104].

Pyrethroids are synthetic chemicals modeled after the structure of the natural insecticidal pyrethrins, derived from chrysanthemum flowers [105]. Synthetic pyrethroids were designed to overcome the undesirable characteristics of pyrethrins, which break down quickly when exposed to air, light, and heat, limiting vector control efficacy [106]. Pyrethroids target and alter the function of voltage-gated sodium channel (VGSC) in the insect nervous system, leading to paralysis and death.

Pyrethroids are classified into the Insecticide Resistance Action Committee Group 3A of sodium channel modulators. Pyrethroids are divided into two types, Type I and Type II [107]. Structurally, the Type I pyrethroids are devoid of the α-cyano group that is characteristic of the Type II group. While both Type I and Type II pyrethroids belong to the same IRAC mode of action group (3A), they interact with VGSCs in distinct ways, resulting in distinct physiological and toxicological effects [108, 109]. One structural exception among pyrethroids is etofenprox, which is a non-ester pyrethroid that, despite lacking the ester bond shares a similar mode of action by acting on sodium channels [110].

Type I pyrethroids can bind to the open state of the VGSC. Their impact on the VGSC involves a prolonged period of channel opening, leading to an extended influx of sodium ions (Na^+^). This results in repetitive neuronal firing, causing tremors known as T syndrome, hyperactivity, followed by paralysis or “knockdown,” leading to a quick death [111].

In contrast, Type II pyrethroids, such as deltamethrin and cypermethrin, include an α-cyano group, which significantly alters their interaction with the VGSC. First developed in 1974, type II pyrethroids preferentially bind to the open state of the VGSC [112–114]. These pyrethroids induce a more pronounced prolonged opening of the VGSC, resulting in a much longer period of influx of sodium ions that can last from several seconds to minutes [115, 116]. This leads to enhanced persistent depolarization of the neuronal membrane, preventing the neuron from resetting and blocking further action potentials. The symptoms caused by Type II pyrethroids are more severe, leading to prolonged paralysis and death of the insect, with initial hyperactivity and convulsions followed by a prolonged state of paralysis [117, 118]. Moreover, the α-cyano group contributes to delayed toxicity, enhancing the potency and persistence of Type II pyrethroids, causing delayed yet more sustained toxic effects compared with Type I pyrethroids [119, 120]. Although their ultimate effects are more profound and lasting, the term “knockdown resistance” (*kdr*) is still broadly applied to resistance mechanisms affecting both Type I and Type II pyrethroids because it describes the initial disruption of neural function that prevents effective insect control, regardless of the subsequent progression to paralysis and death [121].

### Use of synergists

Besides the use of pyrethroids as the active ingredient (a.i.), the inclusion of synergists in their formulation can enhance their effectiveness. Synergists alone, by definition, display low toxicity to most insects [122]. Insecticide synergists are compounds that enhance the efficacy of insecticides by inhibiting the insect enzymes responsible for the degradation of insecticides [123]. Synergists of insecticides typically target cytochrome P450 enzymes (CYP 450s), glutathione-S-transferases (GSTs), or esterases (ESTs). These enzymes are discussed in the “Increased detoxification” section.

The most used commercial insecticide synergist is piperonyl butoxide (PBO; IUPAC: 5-[(2-Butoxyethoxy)methyl]-6-propyl-13-benzodioxole), An inhibitor of CYP 450s [124]. PBO has been commercially used since 1940, mainly in combination with pyrethroid insecticides [125]. Its lack of specificity in CYP 450s inhibition has contributed to its success as a synergist [126].

PBO competitively binds to the active site of CYP 450s and then is metabolized to a semi-irreversible inhibitor complex between a carbene radical of the methylenedioxyphenyl group and the ferrous iron of the CYP 450, inhibiting their activity [127, 128]. By inhibiting these enzymes, PBO prevents the breakdown of insecticides. This inhibition results in a higher concentration of the active ingredient within the insect target site, leading to increased toxicity against insects. Although PBO is considered non-toxic for Humans at the concentrations used in pesticides, there are concerns about its environmental impact. PBO can persist in the environment And potentially affect nontarget organisms by inhibiting their CYP 450s [129]. Recently, however, the concern of resistance to PBO in mosquitoes has emerged, which will further impair mosquito control [130].

In addition to PBO, commercially available synergists include MGK-264 (N-octyl bicycloheptene dicarboximide) [131, 132] And Sesamex, also known as sesoxane, both function by inhibiting CYP 450s [133].

For laboratory studies and diagnosing resistance mechanisms, other synergists employed include S,S,S-tributyl phosphorotrithioate (DEF) and triphenyl phosphate (TPP), which inhibit ESTs [134, 135], and Diethyl Maleate (DEM) and ethacrynic acid (EA) are both used to inhibit GSTs [136, 137].

### Insecticide resistance and resistance mechanisms

Insecticides play a crucial role in preventing disease transmission by targeting arthropod vectors. However, the overuse of these chemicals has led to the development of insecticide resistance across the world, making it increasingly difficult to manage vector populations effectively [138–141]. Phenotypic insecticide resistance is “the development of an ability in a strain of insects to tolerate doses of toxicants that would prove lethal to the majority of insects in a normal population of the same species” [142]. As a result of insecticide resistance, there has been a reduction in the effectiveness of vector control programs [138–141], therefore, insecticide resistance may be a contributing factor to the increase in the number of mosquito-related disease outbreaks in recent years [143–145]. Insecticide resistance can evolve rapidly through selection of preexisting resistance alleles, increasing their frequency in treated populations [146, 147]. This process can result in both cross-resistance (resistance to pesticides with the same mode of action) to similar compounds and multiple resistance (resistance to pesticides with different modes of action) to different insecticide classes.

**Fig.1:**
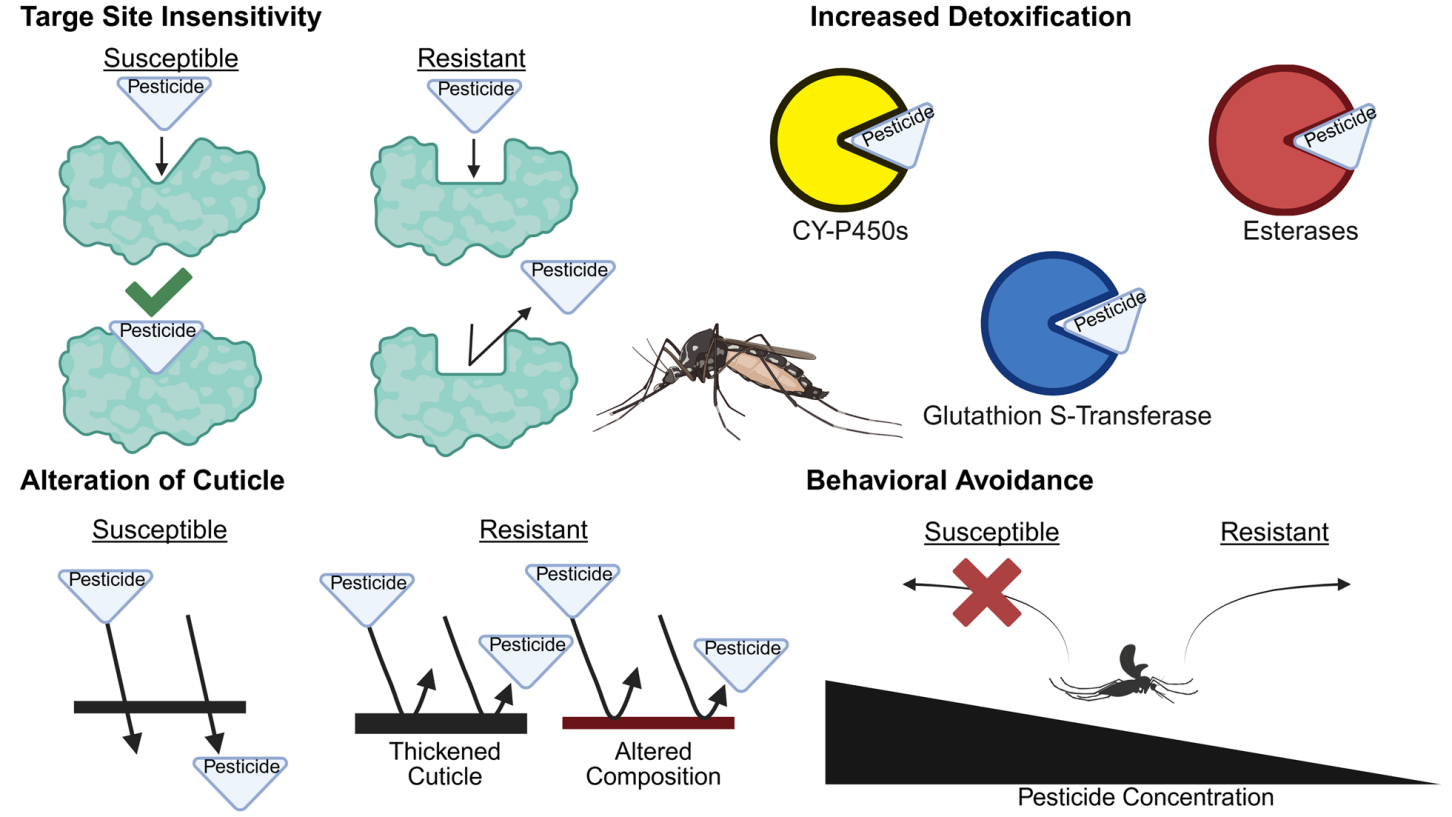
Insecticide resistance mechanisms of *Ae. aegypti*. Target site insensitivity: the target site of the insecticide is altered, preventing the insecticide from effectively binding to it and thereby reducing its efficacy. Increased detoxification: there is an enhanced production of Alterations of the insect cuticle: physiological changes to the cuticle reduce the absorption or penetration of insecticide. Behavioral avoidance: development of behavioral changes that prevent or reduce the negative effects of insecticides

The four main mechanisms of insecticide resistance are target site insensitivity, increased detoxification, reduced penetration, and behavioral avoidance (Fig. 1) [148, 149]. Multiple resistance mechanisms are often involved in individual mosquito species, complicating resistance management strategies [150–152]. Understanding these mechanisms allows for tailored control strategies that account for local resistance profiles, emphasizing the need for molecular surveillance and adaptive management practices.

#### Target site insensitivity

Target site insensitivity occurs when genetic mutations alter the structure of the insecticide’s target site, rendering the insecticide less effective or ineffective [153, 154]. There are several point mutations associated with target site insensitivity documented in *Ae. aegypti* including resistance to dieldrin, or *Rdl,* genes of the gamma-aminobutyric acid (GABA) receptor, point mutations of the nicotinic acetylcholinesterase (*Ace*) receptor, and *kdr* mutations of the VGSC (in *Drosophila melanogaster* the sole VGSC gene is known as *paralytic* (*para))* [153, 155–157]. Occurrence of these mutations are associated with heavy use of their target specific pesticides [158, 159].

In the case of pyrethroids, the target site is the voltage-gated sodium channel. Knockdown resistance (*kdr*) mutations are single-point alterations in the VGSC gene that confer resistance to pyrethroids [160]. The "*kdr*" nomenclature originated from studies on houseflies but has since been widely used in mosquito research [161]. These mutations reduce the binding affinity of pyrethroids, thus diminishing their efficacy and allowing mosquitoes to survive exposure. The role of *kdr* mutations in resistance has been experimentally confirmed through heterologous expression of VGSCs in *Xenopus* oocytes, followed by electrophysiological comparisons, or through congenic strains where resistant mutations have been introgressed into a susceptible genetic background [160, 162]. *Kdr* mutations have been documented in several mosquito species, including *Anopheles gambiae*, *Culex quinquefasciatus*, and *Aedes aegypti*. The spread of these mutations within mosquito populations is facilitated by selective pressure from extensive insecticide use [148, 163, 164]. Consequently, areas with high pyrethroid usage frequently report higher frequencies of *kdr* mutations, reducing the efficacy of control efforts [165–171].

#### Increased detoxification

Enzymatic detoxification is a significant resistance mechanism in insects, including mosquitoes like *Ae. aegypti*, which reduces the toxic effects of insecticides [172, 173]. Overexpression or upregulation of these genes increases the rate at which insecticides are metabolized, which reduces their concentration within the insect’s body, diminishing their effectiveness [104]. A major concern with enzymatic detoxification as a resistance mechanism is that these enzymes can confer broad-spectrum resistance to multiple classes of insecticides, known as “multiple resistance,” not just to pyrethroids [172]. However, there are energetic costs related to enzymatic detoxification that may lead to fitness costs [174, 175]. The primary enzymes involved in this process belong to several families, including CYP 450s, esterases (ESTs), and glutathione S-transferases (GSTs) [176, 177]. In *Ae. aegypti,* genes for 160 CYP 450s, 49 ESTs And 26 ESTs have been identified [178].

CYP 450s are a family of enzymes that catalyze the oxidative metabolism of insecticides, converting them into less toxic or nontoxic metabolites or even more toxic metabolites for insects [176]. Cytochrome P450 enzymes metabolize pyrethroids by oxidation, converting these insecticides into less toxic or more water-soluble compounds. Some CYP 450s that have been found overexpressed in resistant *Ae. aegypti* populations include those of the CYP4, CYP6, and CYP9 families, [179, 180]. A resistant *Ae. aegypti* strain from Singapore, introgressed into the genetic background of the Susceptible Rockefeller strain, helped identified four CYP 450 genes, *CYP6BB2*, *CYP6Z8*, *CYP9M5*, and *CYP9M6* [181], that were associated with pyrethroid resistance through enhanced detoxification. Additionally, several studies have highlighted the involvement of *CYP9J28* in pyrethroid resistance in *Ae. aegypti* [182, 183].

Esterases hydrolyze ester bonds in insecticides, breaking them down into inactive compounds [184]. Elevated esterase activity is often observed in resistant *Ae. aegypti* strains [185]. Esterases detoxify pyrethroids by hydrolyzing ester bonds within the insecticide molecules, effectively neutralizing their toxic effects. An esterase associated with pyrethroid resistance includes *CCEae3a*, which has been observed in transgenic, insecticide-resistant mosquitoes [186–188]. Esterases are also involved in insecticide sequestration, where the insecticide is not metabolized but it is bound to the enzyme, decreasing its effective concentration [189].

GSTs catalyze the conjugation of reduced glutathione to electrophilic compounds, including insecticides [190]. GSTs detoxify pyrethroids by catalyzing the conjugation of a reduced glutathione to the pyrethroid molecules, making them less toxic and easier to excrete. GST-mediated resistance is often linked to the overexpression of specific GSTs genes, such as *GSTD4*, *GSTE2*, and *GSTS1* [179, 191].

Identifying the specific enzymes involved in insecticide resistance in a population is challenging. Although enzymes, Such as CYP 450s, are strongly linked to the development of pyrethroid resistance, the large number of these genes and the structural similarity among different isoforms, makes the identification of isoforms associated with resistance difficult [192]. Additionally, enzyme activity and expression can vary depending on the developmental stage [193], highlighting the importance of routine monitoring for resistance enzymes. Recent studies emphasize that overexpression alone does not fully explain metabolic resistance as factors including allelic variation, gene duplication, and interactions with co-factors like cytochrome P450 reductase (CPR) and cytochrome b5 (CYB5) also influence enzymatic activity and resistance intensity [194].

Understanding these detailed genetic and biochemical factors is crucial for interpreting the broader geographic distribution of insecticide resistance in mosquito populations. Overall, the geographic distribution of metabolic resistance in *Ae. aegypti* as a major factor in insecticide resistance is highly variable, with strong evidence of elevated enzyme expression in South America and Southeast Asia [195]. Recent global comparative genomics work has provided the first large-scale catalog of detoxification gene variants, including those in *GSTE2*, revealing widespread resistance-associated mutations, such as C115F and L111S in populations from Africa, the Americas, and parts of Asia, while these variants were notably absent in Thailand, suggesting regional divergence in resistance mechanisms [35]. However, data are sparse or inconsistent in parts of Africa and Australia, highlighting the need for geographically standardized diagnostic tools to monitor metabolic resistance mechanisms.

Identifying the presence of detoxification enzymes in resistant mosquito populations requires both phenotypic and genotypic approaches. Phenotypic characterization relies heavily on bioassays, where mosquito populations can be segregated into resistant (either field-collected or developed through laboratory selection) and susceptible groups, which are then exposed to either a synergized pyrethroid (e.g., permethrin combined with a synergist like piperonyl butoxide) or a control formulation with only permethrin. Following exposure, or before, for baseline measurements, enzyme activity assays are performed to quantify the activity of key detoxication enzyme classes. The enzyme activity assays utilize substrates such as ethoxyresorufin (for CYP 450s), CDNB (1-chloro-2,4-dinitrobenzene; for GSTs), and α-naphthyl acetate (for esterases) [196]. Additionally, for organophosphate and carbamate resistance, acetylcholinesterase inhibition assays are conducted to detect target-site insensitivity [197]. These tests should be repeated with at least three biological replicates to ensure reliability. To further confirm the functional role of these enzymes in conferring resistance, RNA interference (RNAi) or gene knockout technologies can be used to reduce or eliminate specific enzyme activity, followed by insecticide bioassays to observe changes in resistance levels [198, 199].

Complementing these phenotypic analyses, genotypic characterization involves molecular methods to assess gene expression. RNA is extracted from the collected populations, either from individual mosquitoes or from a pool, and the gene expression levels of detoxification enzymes are measured by quantitative reverse PCR (qRT-PCR), targeting specific resistance-associated genes like *CYP9J32* or *GSTE2*. Additionally, microarray analyses can compare the expression profiles of candidate genes between susceptible–and resistant-strains, identifying enzymes expressed at higher levels [200, 201]. It is crucial to note that the correlation between the transcript And protein levels, particularly for CYP 450s, is not consistently high and relying solely on transcript screens may overlook significant resistance mechanisms that are only detectable at the protein level [202].

In the USA, metabolic resistance in *Ae. aegypti* is understudied. In Florida, bioassays using enzyme inhibitors revealed that most of populations investigated exhibited metabolic resistance Due to either CYP 450s, esterases, and/or GSTs [203–205]. However, the specific expression of these genes was not investigated in these studies. In a separate study conducted in Florida, *CYP6CB1* was upregulated in field-collected *Ae. aegypti* [206]. In California, biochemical assays of *Ae. aegypti* have indicated increased CYP 450 and esterase activity [207, 208].

#### Reduced penetration

##### Alteration of the cuticle

The insect cuticle is composed of chitin, a long-chain polymer of β-(1–4)-*n*-acetyl-*d* glucosamine, along with proteins, lipids, pigments, inorganic materials, and small organic molecules [209]. The cuticle serves as the first line of defense against environmental stresses, including insecticides. However, contact pesticides are designed to penetrate the insect cuticle [210]. Thus, alteration of the insect cuticle is a significant resistance mechanism, albeit less commonly studied [211]. Resistance through cuticular alterations involves changes in the composition, thickness, or structure of the cuticle, which can affect the penetration and absorption of insecticides into the insect’s body [212–214]. Multiple studies have highlighted the alteration of the cuticle in insecticide-resistant mosquitoes [173, 213, 215]. In addition, for *Ae.aegypti*, a species not typically associated with saline environments, mosquitoes reared in brackish water have a thickened cuticle and are more resistance to pesticides including deltamethrin, malathion, permethrin, and etofenprox than mosquitoes reared in freshwater [216].

#### Sequestration by sensory appendage proteins (SAPs)

A more recently identified contributor to resistance includes sensory appendage proteins, particularly *SAP2*, which are located in the mosquito’s legs and olfactory organs and are involved in mosquito navigation and insecticidal avoidance [217]. In *Anopheles gambiae*, overexpression of *SAP2* confers pyrethroid resistance by binding and sequestering insecticides, reducing their penetration [218]. Silencing *SAP2* restores susceptibility to pyrethroids, while its overexpression enhances resistance [218]. Although *SAP2* has been implicated in resistance in *Anopheles*, its role in *Ae. aegypti* remains unclear, as *SAP2* was under expressed in pyrethroid-resistant *Ae. aegypti* from Lahore, suggesting it may not contribute significantly to resistance in that population [182]. However, as this study analyzed whole-body samples, it may have masked tissue-specific differences in gene expression, particularly in the legs or olfactory organs where *SAP2* expression is expected.

#### Behavioral avoidance

“Behavioral resistance” is another less studied but becoming increasingly recognized phenomenon where insects develop adaptive behaviors that reduce their exposure to insecticides [219, 220]. These changes can include temporal, spatial, and trophic shifts to reduce the likelihood of insecticidal exposure. Unlike physiological mechanisms, such as target site insensitivity or enzymatic detoxification, which involve genetic changes within the insect, behavioral “resistance” or tolerance typically focuses on how insects alter their behaviors to avoid or minimize contact with insecticides. [221–223].

Examples of behavioral tolerance include insects exhibiting avoidance behaviors to evade areas treated with insecticides [224]. Alternatively, in response to repeated insecticide exposure but where the hosts are still present in these areas, mosquitoes may alter their activity patterns, such as shifting their peak feeding times or reducing daytime activity when insecticide application is common [225]. Furthermore, opportunistic feeders, such as *Ae. aegypti,*, may feed on hosts that are in non-treated areas [226]. By avoiding treated areas and altering behaviors to minimize contact with insecticides, insects can reduce their exposure to lethal concentrations. A potential behavioral avoidance genetic mechanism has been identified from an odorant-binding protein of *Culex quinquefasciatus* that was correlated with resistance to deltamethrin [227].

### Voltage-gated sodium channels and *kdr* mutations

Pyrethroids are the most globally used insecticides for vector control, targeting the voltage-gated sodium channel (VGSC) [228, 229]. Understanding the VGSC structure and function, along with resistance mechanisms like *kdr* mutations, is crucial for developing effective strategies to combat insecticide resistance, informing the design of new insecticides and resistance management practices to ensure continued efficacy of vector control programs. This section focuses on the VGSC of *Ae. aegypti*, exploring its structure and function, *kdr* mutations, and associated fitness costs.

#### Structure and function of the VGSC

Voltage-gated sodium channels (VGSCs) are integral membrane proteins that play a crucial role in nervous system signaling by initiating and propagating action potentials in neurons. The VGSC is critical in nearly all animals and is thus highly conserved. In humans, the number of known VGSCs includes nine isoforms (Na_v_1.1–Na_v_1.9) encoded in nine different genes, SCN(1–5)A, SCN(8–11)A [230]. The first insect sodium channel gene (the *para* gene, later named *DmNa*_*v*_) was cloned from *Drosophila melanogaster* [231]. In most insects, including *Ae. aegypti*, there is only one sodium channel gene, making these channels the primary targets of pyrethroid insecticides. Advances in understanding the structure of the VGSC come from the high-resolution crystal structure of the potassium channel Kv1.2 of the rat brain and human VGSC [232, 233]. An in-silico model of the housefly sodium channel was constructed based on the crystal structure of the potassium channel [234]. Later, the *Ae. aegypti* VGSC model was predicted based on shared homology with the human VGSC [235, 236].

**Fig.2:**
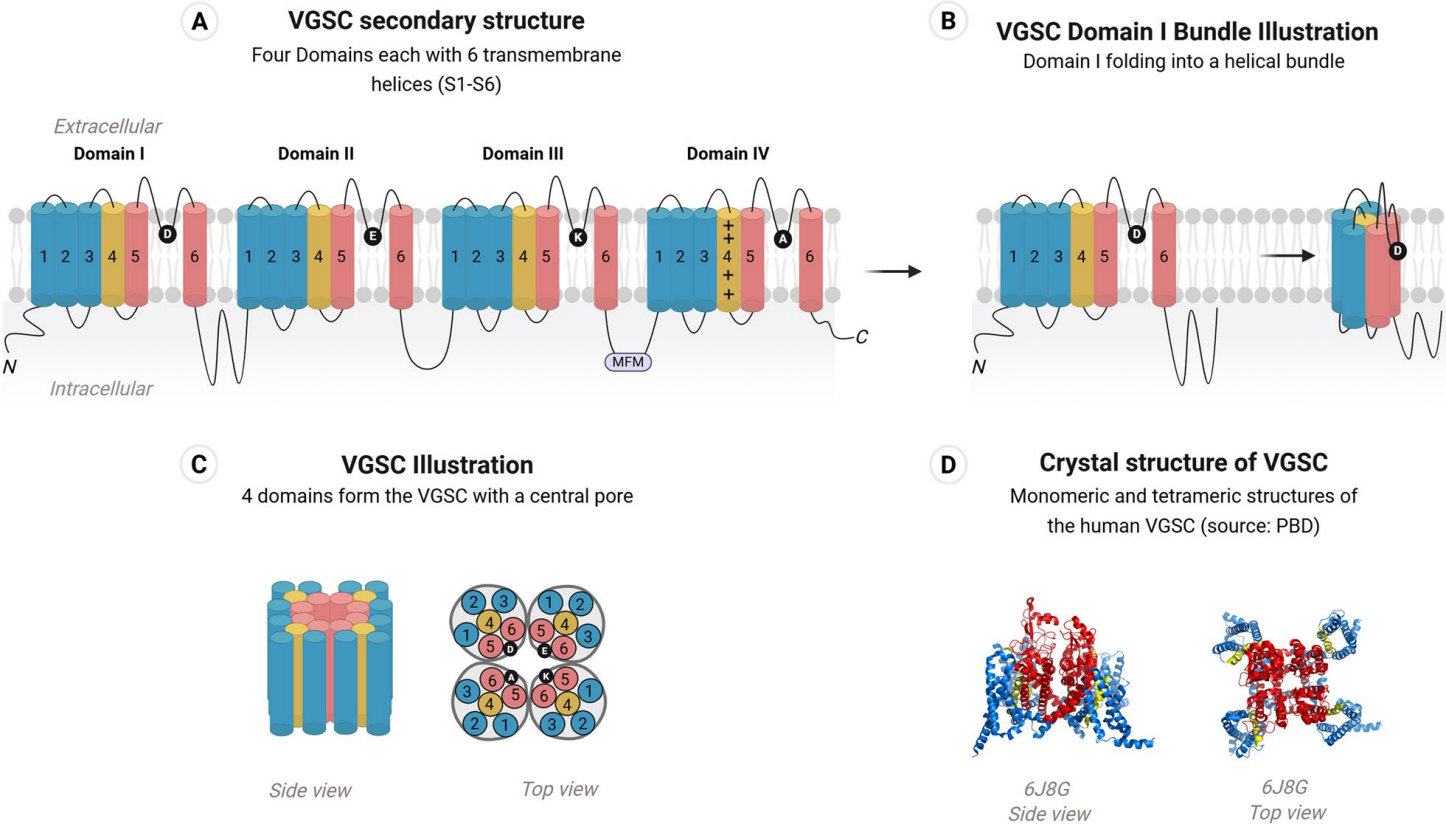
Illustration of the VGSC structure. **A** The secondary structure consists of four domains, each containing six transmembrane alpha helices or segments (S1-S6). **B** Within each domain, these helices fold into a helical bundle, depicted as cylinders for simplicity. **C** The four domains collectively form the voltage-gated sodium channel (VGSC), with a central pore lined by helixes 5 And 6. **D** Side and tope view of the crystal structure of the human VGSC (PBD: 6J8G). The S1-S3 helices, depicted in blue, constitute the outer portion of the voltage-sensing domain [156]. The S4 helices, highlighted in yellow, are the voltage sensors. The S5 and S6 helices in red form the pore domain [367]

The *Ae. aegypti* VGSC consists of a large, single-chain polypeptide alpha subunit (approximately 2050 amino acid residues), comprised of four homologous domains (I–IV), each containing six transmembrane segments (S1–S6; Fig. 2) [162]. Segments S1-S4 form the voltage sensing domain which regulates channel opening upon depolarization of the membrane. Segment S4 acts as the voltage sensor due to positively charged residues, primarily arginine and lysine, occurring every three residues within each S4 helix. Segments S5, S6 and extracellular pore loops (P-loops) connecting S5 and S6 in each domain create the pore domain through which Na^+^ ions pass, containing the selectivity filter. This filter, formed from a hairpin-like loop between segments S5 and S6, ensures ion selectivity and permeation due to the Aspartate (D), Glutamate (E), Lysine (K), and Alanine (A), which form the DEKA motif (Fig. 2) [237].

Within milliseconds after opening, sodium channels undergo fast inactivation. This is mediated by an inactivation particle in the intracellular linker that connects domains III and IV, formed by a MFM (amino acid residues: Methionine, Phenylalanine and Methionine) motif in insect sodium channels and an IFM (amino acid residues: Isoleucine, Phenylalanine and Methionine) motif in mammalian sodium channels [121, 238].

VGSC activation is voltage driven. The S4 is the channel voltage sensor which detects and responds to changes in the plasma membrane potential and, therefore, determines the activity state of the channel [160]. At the cell membrane resting potential of − 70 mV, the VGSC is closed, preventing Na^+^ ions from entering the cell. Upon cell stimulation, if the membrane potential reaches a threshold of −55 mV, the membrane quickly depolarizes to approximately + 40 mV [160]. Upon membrane depolarization, VGSCs open owing to a conformational change initiated by the voltage sensing domain, allowing Na^+^ ions to flow into the cell. This influx generates an action potential, which propagates along the axon, facilitating communication within the nervous system. Within milliseconds, the channels enter a repolarization stage where the inactivation particle occludes the pore by binding to a docking site, preventing Na^+^ from entering the cell. This activation and inactivation is vital for muscle contraction, sensory processing, and overall neurological activity making VGSCs ideal therapeutic targets in mammals and crucial pesticide targets in insects such as *Ae. aegypti* [239, 240].

The insecticidal action of sodium channel modulators (e.g., pyrethroids and dichlorodiphenyltrichloroethane (DDT)) results from their binding and modulation of the VGSCs of insects, altering channel properties and disrupting normal nerve signal transmission [121]. One and two site models have been proposed to model the interaction that pyrethroids have with the VGSC [234, 236]. These receptor sites include pyrethroid receptor site 1 (PyR1) And pyrethroid receptor site 2 (PyR2) [234, 236, 241]. PyR1 includes the S5 segment of domain II, S6 of the II and III domains, and intracellular linker connecting segments S4 and S5 of domain II [234, 241]. The second pyrethroid receptor site, PyR2, consists of the S5 segment of domain I, the S6 segments of the I and II domains, and intracellular linker connecting segments S4 and S5 of domain I [236]. Crucially, *kdr* mutations are frequently identified in these sites, with docking studies supporting that mutations in these sites alter pyrethroid binding by allosterically changing the geometry of the receptor sites, thereby conferring resistance [242]. However, these models do not fully account for how such diverse amino acid changes, even those outside the binding domains, can result in pyrethroid resistance [162].

#### Kdr mutations of *Ae*. *aegypti*

The earliest reports of resistance in insects exposed to pyrethrins And DDT was in the 1950s [243]. Current knowledge supports that resistance to pyrethroids in insects, particularly in mosquitoes such as *Ae. aegypti*, is often associated with mutations in the VGSC [156, 244, 245]. Single-point, non-homologous mutations to the VGSC, known as “knockdown resistance” or *kdr*, at the pyrethroid receptor sites prevent insecticide binding [234]. The molecular basis of *kdr* mutations in insects was discovered in 1996 in *Musca domestica*, where pyrethroid resistance was linked to point mutations (L1014F in the S6 segment and M918T in the S4–S5 linker, both within domain II [246]. Since then, *kdr*-like mutations at the 1014-site (L to F/H/S mutation in IIS6) have been reported in various insect species including mosquitoes [247–250]. *Kdr* mutations can occur individually or simultaneously, with different expression of resistance [162]. Specific mutations have been identified, such as L1014F, V1016G/I, and F1534C, which are prevalent in diverse mosquito populations worldwide (Fig. S1).

**Fig.3:**
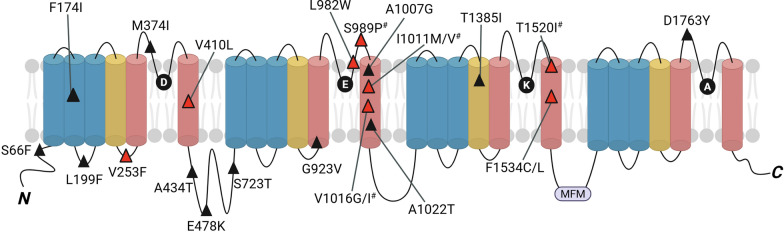
Nonsynonymous mutations in the voltage-gated sodium channel of *Ae. aegypti*. Mutations with red triangles denote mutations that have been functionally confirmed to reduce sensitivity to pyrethroids. Mutations with black triangles have been reported in pyrethroid-resistant *Ae. aegypti* but have not yet been functionally supported. Mutations with a pound symbol (^#^) do not independently reduce sensitivity to pyrethroids but synergistically increase resistance when combined with other functional *kdr* mutations [235, 236]

In *Ae. aegypti*, single-point, non-homologous mutations have been identified in resistant populations throughout the world (Fig. 3; Table 2). The geographic distribution, frequency, and co-occurrence of these mutations can vary greatly [251]. Furthermore, these mutations have not all been confirmed to decrease sensitivity to pyrethroids. Single *kdr* mutations of *Ae. aegypti* related to pyrethroid resistance that have been confirmed in electrophysiological studies include: V253F [252], V410L [253], L982W [254], I1011M [236], V1016G [236, 255], and F1534C [236]. Interestingly, *Aedes aegypti* does not exhibit the highly pyrethroid-resistant *super-kdr* genotype, characterized by the co-occurrence of the L1014F and M918T mutations. This may be due to the Substitution at residue 1014 in the *Ae. aegypti* sodium channel requiring two base-pair changes to code for phenylalanine (F), and the M918T mutation has not been observed in the absence of the L1014 mutation in mosquitoes [162]. However, *Ae. aegypti* frequently develops high levels of pyrethroid resistance through the co-occurrence of other *kdr* mutations, with distinct sets of genotypes that confer higher prevalence and increased intensity of resistance such as L982W + F1534C [256], V1016I + F1534C [257], S989P + V1016G + F1534C [258], L199F + A434T + L982W + F1534C [256], V410L + V1016I + F1534C [169]. The sodium channel haplotype S989P + V1016G + F1534C when expressed in *Xenopus* oocytes, reduced sensitivity by 1100-fold against permethrin And by 90-fold against deltamethrin [255].

**Table 2:** Mutations in the voltage-gated sodium channel of *Aedes aegypti* associated with pyrethroid resistance and countries where they were found

Mutation	Original amino acid	Mutant amino acid	Countries found	Reduces functional sensitivity to pyrethroids
*S66F (S76F)*	Serine	Phenylalanine	Vietnam and Cambodia [256]	–
*F174I (F184I)*	Phenylalanine	Isoleucine	USA (Florida), [368]	–
*L199F (L209F)*	Leucine	Phenylalanine	Vietnam and Cambodia [256]	–
***V253F ****(V263F)*	Valine	Phenylalanine	Brazil [265]	Yes (Type I and II) [252]
*M374I (M383I)*	Methionine	Isoleucine	Brazil [265], Cambodia and Vietnam [256]	-
***V410L ****(V419L)*	Valine	Leucine	USA [170], Brazil [253], Mexico [369], Africa [370], Colombia [371], Argentina [372]	Yes (Type I and II) [253]
*A434T (A443T)*	Alanine	Threonine	Vietnam and Cambodia [256]	–
*E478K (E476K)*	Glutamic Acid	Lysine	USA (Florida) [368]	–
*S723T (S694T)*	Serine	Threonine	USA (California), Vietnam and Cambodia [256], Mexico [373, 374]	–
*G923V (G902V)*	Glycine	Valine	Brazil, Guyana, and Martinique [269]	–
***L982W ****(L961I)*	Leucine	Tryptophan	Vietnam and Cambodia [256]	Yes (Type I and II) [254]
*S989P*^*#*^* (S968P)*	Serine	Proline	Africa [375, 376], Multiple Asian countries [270], USA (Florida) [272]	No^*#*^ [236, 255]
*A1007G (A986G)*	Arginine	Glycine	Malaysia [377]	–
***I1011M ****(I990M)*	Isoleucine	Methionine	Brazil [378], Mexico [319]	Yes (Type I only) [236]
*I1011V*^*#*^* (I990V)*	Isoleucine	Valine	Brazil [378], Mexico [319]	–
*V1016I*^*#*^* (V995I)*	Valine	Isoleucine	USA [171, 333], Argentina [379], Colombia [380], Venezuela [381], Mexico [378], Grand Cayman [382], Vietnam [383], Martinique [150]	No^*#*^ [236]; Reduces sensitivity to DDT [235]
***V1016G ****(V995G)*	Valine	Glycine	Majority of Asia [259], Africa [375], USA (Florida) [272]	Yes (Type I and II) [236]
*A1022T(A1001T)*	Alanine	Threonine	India [384]	–
*T1385I (T1375I)*	Threonine	Isoleucine	Vietnam and Cambodia [256]	–
*T1520I*^*#*^* (T1510I)*	Threonine	Isoleucine	India [385], Myanmar [386]	No^*#*^ [235]
***F1534C ****(F1524C)*	Phenylalanine	Cysteine	Most Continents [263]	Yes (Type I only) [236, 387]
*F1534L (F1524L)*	Phenylalanine	Leucine	India [388]	–
*D1763Y (D1753Y)*	Aspartic Acid	Tyrosine	Taiwan [389]	No^*#*^ [236]

*Kdr* mutation nomenclature specifies the amino acid change at specific positions of the amino acid sequence of the *Musca domestica* sodium channel (GenBank accession number: AAB47604). In parentheses are the amino acid position numbers from the VGSC of *Ae. aegypti* (GenBank accession number: AGG53064)

*Kdr* mutations functionally confirmed in *Xenopus* oocytes as impairing pyrethroid action are in bold. Mutations with a number symbol (^a^) do not independently reduce sensitivity to pyrethroids, but when co-occurring with other *kdr* mutations, these mutations enhance resistance

The most globally distributed *kdr* genotype in *Ae. aegypti* is F1534C (Phenylalanine to Cysteine) in Domain III, S6 [259]. Australia is the only continent where this mutation has not been detected [260]. When this mutation is expressed alone in congenic mosquitoes, it confers resistance to type-I (permethrin) and type-II (deltamethrin) pyrethroids [261]. However, electrophysiological studies in the *Xenopus* system have revealed that this mutation confers reduced VGSC sensitivity to permethrin, but not to deltamethrin [236]. This discrepancy between in vivo phenotypic resistance observed in *Ae. aegypti* and in vitro electrophysiological findings in a heterologous expression system highlights the complexity of functional expression in heterologous systems to study *kdr* resistance mechanisms. In bioassays with congenic mosquitoes, every aspect of the intoxication process is evaluated, from penetration, detoxification and finally the effect at the VGSC target, while the heterologous system only provides information on target sensitivity [162]. While F1534C clearly contributes to permethrin resistance, its role in deltamethrin resistance may be more nuanced. Further research is needed to reconcile these findings and fully elucidate the contribution of F1534C to pyrethroid resistance profiles in natural *Ae. aegypti* populations.

The F1534C genotype is frequently associated with V1016I (Valine to Isoleucine) in domain II, S6. Both mutations are common in highly resistant *Ae. aegypti* populations across the world, with over 90% frequency in some municipalities in Central and South America, likely due to strong selection caused by repeated pyrethroid use [167, 170, 171, 262–264]. While V1016I alone does not alter sensitivity to pyrethroids in *Xenopus* oocytes [236], its co-occurrence with other mutations has been associated with synergistic resistance, where cumulative effects lead to enhanced resistance beyond their individual contributions [265, 266]. It is also possible that the 1016-site mutation reduces the fitness cost of mosquitoes carrying other *kdr* mutations in the VGSC when under selection pressure [267]. Developing strains of *Ae. aegypti* expressing only the V1016I mutation may in the future provide valuable insight into the role of this mutation.

Other mutations in the voltage gated sodium channels of *Ae. aegypti* associated with pyrethroid resistance include V410L (Valine–Leucine), S989P (Serine–Proline), I1011M (Isoleucine–Methionine), and V1016G (Valine 1016–Glycine) [253, 268, 269]. These mutations have been observed in specific geographic regions and the co-occurrence of specific mutations are associated with further increased levels of insecticide resistance. Interestingly, the V1016G mutation alone confers moderate levels of insensitivity to type-I and type-II pyrethroids, while V1016I does not [236, 255]. V410L is among the more recently observed of these mutations which was first discovered in Brazil but has been found in other countries [169, 170, 253, 264, 270]. Heterologous expression in *Xenopus* oocytes has shown that the V410L *kdr* mutation alone decreases the VGSC’s sensitivity to both type-I and type-II pyrethroids [253].

Although mutations, such as S989P and V1016G, are strongly linked to their geographic locales in both Asia and Oceania and are considered regional, there are concerns of mosquitoes with resistant genotypes migrating to other countries via commercial aircrafts and ships [271]. Recently, *Ae. aegypti* with the V1016G and S989P resistant mutations were detected for the first time in the US in Florida [272]. It is currently unclear how the interaction of these mutations with the V410L, V1016I, and F1534C mutations will have on current operational interventions to abate mosquito populations. The discovery of *kdr* mutations in the USA previously found on other continents demonstrates the urgent need for continuous monitoring, resistance screening, and novel strategies to manage mosquito populations while mitigating the spread of insecticide resistance. Comparative analysis of pyrethroid resistance trends across regions provides valuable insight into the global spread of resistance alleles, as does the screening for new mutations discovered in other species [273].

### Detection of insecticide resistance

Detecting insecticide resistance in insect populations is critical for effective pest management as field insecticide resistance can lead to the failure of control programs [274]. Understanding and monitoring resistance patterns helps in developing strategies to mitigate these risks. To do this, there are a variety of methods to estimate insecticide resistance [275] which include field bioassays, laboratory bioassays, and molecular and biochemical analyses to identify resistance genes and overexpressed enzymes present in a population [196, 276, 277]. Bioassays can determine the mortality rates, resistance frequency and level of the insect population at a particular time point.

The mosquito bioassays and molecular techniques to monitor resistance should utilize a susceptible strain as a control group [278, 279]. Among the most well-studied pesticide-susceptible strains of *Ae. aegypti* are Orlando [280], Liverpool [281], Rockefeller [282], and New Orleans [186] strains. A lower-than-expected mortality rate in phenotypic bioassays compared with susceptible controls indicates potential resistance. However, even among these susceptible strains, it is important to note that there are differences in their level of susceptibility to different pesticides [283].

#### Applied bioassays

Applied bioassays assess insecticide efficacy under realistic environmental conditions, rather than providing a direct measure of physiological insecticide resistance. These are categorized into operational, field, and semi-field tests [20]. Operational bioassays are conducted as part of mosquito control operations. Field bioassays are conducted in open air, unconfined conditions, while semi-field bioassays are conducted outdoors, but typically in a closed, controlled environment, such as within larger enveloping cages, for safety and/or to avoid disruptive environmental factors that prevent field tests. Applied bioassays typically involve applying insecticides either through space spraying (fogging or ultra-low volume spraying), applying spatial repellents, or treating surfaces such as walls or bed nets where mosquitoes are likely to come into contact indoors [284–286].

However, field and mosquito conditions, including factors such as temperature, humidity, wind speed, and mosquito age, can influence results and complicate interpretation [287–289]. Furthermore, the setup of field and semi-field tests can vary significantly based on the vector species, delivery method, and available resources [274]. This includes considerations such as indoor versus outdoor environments, backpack spraying versus truck-mounted sprayers, pesticides (or repellents) used, and the use of subsample cages versus true replicates [286]. Conducting field assays can be costly and labor-intensive [290]. Quantifying the exact amount of insecticide deposited is also challenging, and uneven insecticide distribution may falsely suggest resistance [250, 291–293].

When interpreting results from these tests, it is critical to distinguish the relationship between adequate insecticide efficacy and absence of insecticide resistance. While field assays may demonstrate product effectiveness, this efficacy can stem from heightened performance owing to synergists, surfactants, or formulation additives that are included in the product [294]. Simply observing high mortality under field conditions does not exclude resistance, and such erroneous conclusion could lead to delayed or inadequate resistance management. Given these challenges, field bioassays, while essential for assessing the real-world effectiveness of insecticides, should be complemented with laboratory tests and continuous resistance monitoring [295, 296]. Further, improvements in techniques such as more accurate dosimetry tools and behavioral studies enhance reliability and interpretability [297].

#### Laboratory bioassays

Laboratory-based bioassays provide controlled environments to assess insecticide susceptibility more precisely and assist in developing effective vector control strategies and mitigating the impact of resistance on public health interventions [104, 298, 299]. By comparing mortality rates across multiple concentrations, researchers can establish dose–response relationships that help identify potential resistance [300]. Alternatively, diagnostic concentrations of insecticides can quickly and efficiently identify resistant populations in laboratory settings [301]. These tests some degree of flexibility by assessing resistance through the use of synergists (e.g., PBO) to determine the role of enzymatic detoxification in resistance [204]. The most used insecticide susceptibility bioassays for mosquitoes include the Centers for Disease Control and Prevention (CDC) bottle bioassay, World Health Organization (WHO) tube test, WHO bottle bioassays, and topical assays [302].

##### CDC bottle bioassay

Developed by the Centers for Disease Control and Prevention (CDC), the CDC bottle bioassay is a standard method for assessing insecticide susceptibility in mosquitoes [303–305]. In the bottle bioassay, a bottle is coated with diagnostic concentration of An insecticide. Mosquitoes are then placed in the treated bottle, and mortality is recorded at 5-min intervals for the first 15 min, And then at 15-min intervals thereafter, for a total exposure period of two hours. Assays are carried out for the full two hours, or until 100% mortality is achieved. Resistance is then determined by the percentage of mosquitoes that died at the diagnostic time, following WHO recommendations [301]. The diagnostic time is the expected exposure Duration required for this diagnostic dose to achieve 100% mortality in a Susceptible population. A mortality rate above 97% indicates pesticide Susceptibility. A mortality rate between 90 And 96% Suggests the population is developing resistance. If the mortality rate is below 90%, it indicates that the population is resistant to a pesticide. CDC bottle bioassays can also be modified to generate dose response data [306]. The CDC bottle bioassay allows for rapid screening of resistance and can be adapted for various insecticides and concentrations [304]. However, variability in insecticide coating, bottle preparation, and mosquito characteristics (e.g., age, sex, and genetic background) can influence reproducibility and outcomes [307–312].

##### WHO tube test

The WHO tube test is a standard method for assessing insecticide susceptibility in mosquitoes [313]. In this assay, mosquitoes are exposed to diagnostic concentrations of insecticide-treated papers Lining tubes where they are confined for a specified period, typically An hour. The insecticide-treated paper is then removed, and mortality is assessed after 24 h. Although this method is simple to execute, its reliance on a limited range of formulations and the requirement for F0 or F1 lab-reared adults can constrain its application, particularly in resource-limited settings [142].

##### WHO bottle bioassays

The WHO bottle bioassay is a variation of the CDC bottle bioassay where glass or plastic bottles are coated with diagnostic concentrations of an insecticide [142]. Mosquitoes are then placed into these bottles for an hour, afterwards transferred into a clean, insecticide-free container, and their mortality is observed over a specific Duration, typically 24 h. This method is like the WHO tube test but allows for the use of different insecticide formulations that are not available as impregnated papers, such as chlorfenapyr.

##### Topical assay

The topical assay is a technique where a precise amount of a technical grade insecticide dissolved in a nontoxic and volatile solvent, such as acetone, is directly applied to the mosquito’s cuticle, usually on the thorax, using a microapplicator, after immobilization on cold trays or CO_2_ exposure [314]. After insecticide application mosquitoes are confined for a specified time (e.g., 24 h). The topical assay offers a critical advantage in enabling dosimetry. This method ensures that an exact amount of insecticide is delivered to each mosquito, allowing for a high degree of precision in determining the intrinsic activity of an insecticide and directly quantifying a dose–response relationship. This precision helps prevent complications from behavioral avoidance and accounts for the variability in exposure that can occur in assays where mosquitoes contact a treated surface, such as in bottle bioassays where the precise amount of insecticide each mosquito acquires is unknown. The topical assay yields dose–response curves, which estimate crucial variables including the median knockdown dose (KD_50_) or the median lethal dose (LD_50_) or the dose that kills most (90%) individuals (LD_90_). The KD_50_ represents the dose (typically given in nanograms per mosquito (ng/mosquito), micrograms per mosquito (µg/mosquito), or as nanograms per milligram of mosquito body weight (ng/mg)) which knocks down 50% of the tested mosquito population within a specific period. Similarly, the LD_50_ indicates the dose (expressed in the same units as KD_50_) that results in the death of 50% of the population, normally in 24 h. Criticisms of topical assays include the labor-intensive nature of the assay and the requirement for skilled personnel to administer precise doses, which may limit its use in routine surveillance [315]. Additionally, mosquito stress from handling may affect their survival, potentially confounding the results [316].

##### Molecular techniques and enzyme activity tests to assess resistance

In addition to bioassays, molecular techniques can identify specific resistance mutations in target genes (e.g., *kdr* mutations) associated with insecticide resistance. Molecular techniques to identify *kdr* mutations include obtaining PCR products of segments of the target site where mutations related to insecticide resistance occur, followed by sequencing, or allele-specific PCR, which identifies mutations based on the sizes of PCR products [317, 318]. There are published allele-specific primers that can be used to identify *kdr* mutations of *Ae. aegypti* populations [253, 319–322]. Furthermore, single nucleotide polymorphism (SNP) genotyping, often performed using high-throughput platforms, provides a rapid and efficient method for detecting known resistance-associated mutations, including *kdr* mutations and detoxification enzymes, across a large number of samples [263, 323, 324]. These platforms commonly utilize technologies such as quantitative PCR-based assays (e.g., TaqMan) or next-generation sequencing to rapidly identify specific nucleotide variations associated with resistance. Assessing the activity levels of detoxifying enzymes (e.g., CYP 450s, ESTs, GSTs) in insect populations can indicate enhanced detoxification capability, a common resistance mechanism [178]. While these molecular and biochemical approaches provide evidence of potential resistance mechanisms, the presence of resistance-associated genes does not necessarily confirm functional resistance [325, 326]. Indeed, molecular methods primarily detect known resistance mutations and may not identify novel resistance mechanisms or confirm functional resistance. Therefore, bioassays are essential to assess if a population is phenotypically resistant. The involvement of metabolic enzymes should also be investigated and inferred through phenotypic bioassays by incorporating synergists, where the restoration of susceptibility when exposed to a synergist links the corresponding enzyme class to resistance in that population [327].

### Resistance levels of *Ae. aegypti* to permethrin and deltamethrin

A comprehensive understanding of the resistance levels of *Ae. aegypti* to permethrin (a Type I pyrethroid) and deltamethrin (a Type II pyrethroid), the two most widely used pyrethroids in vector control, is crucial for assessing the effectiveness of current control strategies and identifying emerging resistance trends [328, 329]. Table 3 shows resistance ratios (RR) among *Ae. aegypti* populations that were treated with either permethrin or deltamethrin, from different geographical regions, to assess patterns and underlying resistance mechanisms in each population, distinguishing between field-collected and resistant laboratory-maintained strains. The extent of pyrethroid resistance in *Ae. aegypti* populations varies substantially, with populations considered susceptible (resistance ratios, RR: less than 5), moderate resistance (RR: 5–10), and high resistance (RR: greater than 10) [330].

**Table 3:** Permethrin and Deltamethrin Resistance Ratios for *Aedes aegypti* Mosquito Strains Across Geographical Locations

Insecticide	Mosquito Source	Country of Origin	*Ae. aegypti* populations/strains tested	Susceptible Strain Used	Life Stage	Method	Toxicological Metrics	RR*	Resistance Mechanism Investigated?	Reference
D	Laboratory	Mexico	Merida, Mexico	New Orleans	Adult	CDC bottle bioassay	KC_50_ (µg/bottle)	2.0 to 38.0	*Kdr* mutations (V410L, V1016I, F1534C) and enzymatic activity	[390]
D	Laboratory	Mexico	(Merida, Hunucma, and Jose Cardel), Mexico	New Orleans	Adult	CDC bottle bioassay	LC_50_ (µg/bottle)	6.0 to 193.0	Enzymatic activity, cuticle, immunity (from transcriptomic analysis)	[173]
D	Laboratory	Cuba	SAN F13: a propoxur-resistant strain	Rockefeller	Larval	WHO larval bioassay	LC_50_ (µg/mL)	26.25	Enzymatic activity	[193]
D	Laboratory	Cuba	SAN-F6: a temephos-resistant strain	Rockefeller	Larval	WHO larval bioassay	LC_50_ (µg/mL)	41.25	Enzymatic activity	[193]
D	Laboratory	Cuba	SAN F12: deltamethrin-resistant strain	Rockefeller	Larval	WHO larval bioassay	LC_50_ (µg/mL)	225	Enzymatic activity	[193]
D	Laboratory	Cuba	Santiago de Cuba, Cuba	Rockefeller	Adult	Bottle bioassay	LC_50_ (mg/L)	4.75 to 337.5	Enzymatic activity	[391]
D	Laboratory	Cuba	Santiago de Cuba, Cuba	Rockefeller	Adult	WHO bioassay	LC_50_ (mg/L)	113.7 to 1425	Metabolic detoxification—Synergism Bioassays (PBO or DEF) and Enzymatic activity	[392]
D	Laboratory	Cuba	Havana City, Cuba	Rockefeller	Larval	Larval Bioassay	LC_50_ (mg/L)	100 to 112.5	Enzymatic activity	[152]
D	Laboratory	French West Indies	Vauclin Strain	Bora-Bora	Adult	WHO tube assays	LD_50_ (% a.i. /paper)	32	N/A	[393]
D	Laboratory	French West Indies	Vauclin Strain	Bora-Bora	Adult	Topical Application	LD_50_ (µg/L)	56	Metabolic detoxification (Synergism Bioassays; PBO, DEF, DMC)	[394]
D	Laboratory	Jamaica	Jamaica	Rockefeller	Larval	Larval Bioassay	LC_50_ (mg/L)	3.7	Enzymatic activity	[152]
D	Laboratory	Nicaragua	Nicaragua	Rockefeller	Larval	Larval Bioassay	LC_50_ (mg/L)	0.4	Enzymatic activity	[152]
D	Laboratory	Panama	Panama	Rockefeller	Larval	Larval Bioassay	LC_50_ (mg/L)	2.5	Enzymatic activity	[152]
D	Laboratory	Puerto Rico	Pyrethroid-resistant Puerto Rican strain (PR)	Orlando	Adult	Topical Application	LD_50_ (ng/mg of mosquito)	650	N/A	[395]
D	Laboratory	Puerto Rico	PR after 20 generations of permethrin selection	Orlando	Larval	Larval Bioassay	LC_50_ (ppm)	17,000	Metabolic detoxification (Synergism Bioassays; PBO, DEM, or DEF)	[205]
D	Laboratory	Brazil	MCNaeg-C (Possesses V253F, M374I, G923S, and F1534C *kdr* alleles)	SMK (Susceptible reference strain from Sumitomo Chemical Co., Ltd. in 2009)	Adult	Topical Application	LD_50_ (ng/mosquito)	64	*Kdr* mutations (V253F, M374I, V410L, S723T, G923S, V1016I, F1534C)	[265]
D	Laboratory	Brazil	MCNaeg-LIC (Possesses V410L, S723T, V1016I, and F1534C *kdr* alleles)	SMK	Adult	Topical Application	LD_50_ (ng/mosquito)	43	*Kdr* mutations (V253F, M374I, V410L, S723T, G923S, V1016I, F1534C)	[265]
D	Laboratory	Peru	Peru	Rockefeller	Larval	Larval Bioassay	LC_50_ (mg/L)	87.5	Enzymatic activity	[152]
D	Laboratory	Venezuela	Venezuela	Rockefeller	Larval	Larval Bioassay	LC_50_ (mg/L)	16.2	Enzymatic activity	[152]
D	Laboratory	France	Ile Royale, French Guiana (IR 13)	New Orleans	Adult	WHO bioassay	KT_50_ (min)	6.42	*Kdr* mutations (V1016I, F1534C) and detoxification gene expression	[396]
D	Laboratory	France	Ile Royale, French Guiana (IR 03)	New Orleans	Adult	WHO bioassay	KT_50_ (min)	51.6	*Kdr* mutations (V1016I, F1534C) and detoxification gene expression	[396]
D	Laboratory	Central African Republic	Institut Pasteur de Bangui, Bangui, African Republic	Rockefeller	Adult	WHO bioassay	LC_50_ (mg/L)	1 to 1.7	Detoxification enzymes	[397]
D	Laboratory	India	DAS20, Delhi, India	Unspecified	Adult	WHO bioassay	LT_50_ (min)	2.6 to 3.8	Behavioral (contact irritability)	[398]
D	Laboratory	India	Deltamethrin selected strain from International Centre for Genetic Engineering and Biotechnology, New Delhi, India	Parent susceptible strain	Larval	WHO larval bioassay	LC_50_ (mg/L)	0.71 to 9.91	Cuticular Thickening	[399]
D	Laboratory	Thailand	Khu Bua, Thailand	New Orleans	Adult	WHO bioassay	KT_50_ (min)	3.88 to 11.2	*Kdr* mutations (S989P, V1016G)	[268]
D	Laboratory	Indonesia	Aceh, Indonesia	Bora-Bora	Adult	WHO bioassay	KT_50_ (min)	2.56 to 72.2	Metabolic detoxification (Synergism Bioassays; PBO or DEF)	[400]
D	Laboratory	French Polynesia	Paea, Tahiti, French Polynesia	Rockefeller	Larval	WHO larval bioassay	LC_50_ (mg/L)	0.8	N/A	[401]
D	Field	USA	St. Augustine, Florida, USA	Orlando	Larval	Larval Bioassay	LC_50_ (ppm)	4.5	Metabolic detoxification (Synergism Bioassays; PBO, DEM, or DEF)	[205]
D	Field	USA	St. Augustine, Florida, USA	Orlando	Adult	CDC bottle bioassay	LD_50_ (ng/mg)	12.6–56.8	*Kdr* mutations (V1016I, F1534C) and enzymatic activity	[166]
D	Field	Mexico	Panuco, Veracruz, Mexico	New Orleans	Adult	Bottle bioassay	LC_50_ (µg/bottle)	0.11 to 19.44	N/A	[402]
D	Field	Mexico	San Luis Acatlán, Guerrero, Mexico	New Orleans	Adult	CDC bottle bioassay	LC_50_ (mg/L)	0.33 to 1.2	N/A	[403]
D	Field	Mexico	Acapulco, Guerrero, Mexico	New Orleans	Adult	Bottle bioassay	LC_50_ (µg/bottle)	0.04 to 9.63	*Kdr* mutations (V1016I, F1534C)	[404]
D	Field	Costa Rica	Guacimo, Costa Rica	Rockefeller	Larval	Larval Bioassay	LC_50_ (mg/L)	1.43	Metabolic detoxification (Synergism Bioassays; PBO and DEF)	[405]
D	Field	Brazil	Rio de Janeiro, Brazil	Rockefeller	Adult	WHO tube assays	LC50 (mg/m^2^)	33.1	*Kdr* mutations (V1016I, F1534C) and enzymatic activity	[83]
D	Field	Brazil	Duque de Caxias, RJ, Brazil	Rockefeller	Adult	WHO bioassay	LC_50_ (mg/m^2^)	6.3 to 70.9	*Kdr* mutations (I1011M, V1016I, F1534C) and enzymatic activity	[406]
D	Field	Brazil	Boa Vista	Rockefeller	Adult	WHO bioassay	LC_50_ (mg/m^2^)	9.4 to 47.4	N/A	[145]
D	Field	Brazil	Aracaju, SE, Brazil	Rockefeller	Adult	WHO bioassay	LC_50_ (mg/m^2^)	14.3 to 37	*Kdr* mutations (V1016I, F1534C) and enzymatic activity	[407]
D	Field	Colombian Caribbean Region	Puerto Colombia, Atlántico	Rockefeller	Adult	CDC bottle bioassay	KC_50_ (µg/bottle)	1.3 to 41.3	*Kdr* mutations (V1016I) and enzymatic activity	[408]
D	Field	France	Petite Terre, Mayotte	Rockefeller	Adult	WHO bioassay	KT_50_ (min)	1.03	Enzymatic activity	[354]
D	Field	Nigeria	Bayero University, Kano, BUK, Nigeria	New Orleans	Larval	Larval Bioassay	LC_50_ (mg/L)	2.25 to 24.75	*Kdr* mutations (V1016G, F1534C) and metabolic detoxification (Synergism Bioassays; PBO or DEF)	[409]
D	Field	West Africa	National Zoological Park, Abidjan, Côte d’Ivoire, West Africa	Bora-Bora	Adult	WHO bioassay	KT_50_ (min)	1.18 to 1.68	N/A	[410]
D	Field	West Africa	Adobo, Abidjan, Côte d’Ivoire, West Africa	Benin (Susceptible Strain)	Adult	WHO bioassay	KT_50_ (min)	1.8 to 3.25	*Kdr* mutations (V410L, V1016I, F1534C)	[411]
D	Field	Pakistan	Faisalabad, Pakistan, Urban	Susceptible laboratory strain collected from Bahawalpu, Pakistan	Adult	WHO (Contact)	LC_50_ (ppm)	1.32 to 19.14	Enzymatic activity	[412]
D	Field	Pakistan	Multan, Punjab, Pakistan	Unspecified	Larval	WHO larval bioassay	LC_50_ (µg/mL)	26.68 to 43.26	N/A	[413]
D	Field	Pakistan	Mishri Shah, Lahore District, Punjab, Pakistan	Government College University, Lahore Susceptible Strain	Adult	CDC bottle bioassay	LT_50_ (min)	1.9	N/A	[414]
D	Field	Pakistan	Faisalabad, Punjab, Pakistan	Susceptible Lab Strain Collected Indigenously	Adult	CDC bottle bioassay	KC_50_ (µg/mL)	2.32 to 18.7	Metabolic detoxification (Synergism Bioassays; PBO or DEF)	[415]
D	Field	Pakistan	Mishri Shah, Lahore District, Punjab, Pakistan	Unspecified	Adult	WHO bioassay	LC_50_ (% a.i./ paper)	10.0 to 20.0	Enzymatic activity	[416]
D	Field	India	Basaveshwara nagar, India	Unspecified	Larval	WHO larval bioassay	LC_50_ (mg/L)	1 to 6.14	N/A	[417]
D	Field	Singapore	Jurong East, Singapore	Bora-Bora	Adult	WHO bioassay	KT_50_ (min)	68.5 to 136.8	Detoxification enzymes	[418]
D	Field	Taiwan	Annan, Taiwan	Gainesville Strain (From USDA)	Adult	WHO bioassay	KT_50_ (min)	1.73 to 88.64	N/A (Behavioral responses)	[419]
D	Field	Thailand	Amnat Charoen, Thailand	Bora-Bora	Adult	WHO tube assays	LT_50_ (min)	13.95 to 24.8	Enzymatic activity	[420]
D	Field	Thailand	Bang Khen, Bangkok, Thailand	Bora-Bora	Adult	WHO bioassay	LT_50_ (min)	8 to 17.2	*Kdr* mutations (V1016G-absent) and enzymatic activity	[421]
D	Field	Thailand	Ladlumkaew Pathum Thani, Thailand	Bora-Bora	Adult	WHO bioassay	LT_50_ (min)	10.4	*Kdr* mutations (V1016G-absent) and enzymatic activity	[421]
D	Field	Malaysia	Hulu Langat, Selangor, Malaysia	Bora-Bora	Adult	CDC bottle bioassay	KT_50_ (min)	1.18	*Kdr* mutations (S989P, V1016G, F1534C) and metabolic detoxification—Synergism Bioassays (PBO, EA, or DEF) and Enzymatic activity	[331]
D	Field	Malaysia	Gombak, Selangor, Malaysia	Bora-Bora	Adult	CDC bottle bioassay	KT_50_ (min)	0.6 to 249.3	*Kdr* mutations (S989P, V1016G, F1534C) and metabolic detoxification—Synergism Bioassays (PBO, EA, or DEF) and Enzymatic activity	[331]
D	Field	Malaysia	Penang Island, Malaysia	Institute for Medical Research, Malaysia Susceptible Laboratory Strain	Adult	WHO bioassay	KT_50_ (min)	3.17 to 3.66	Metabolic detoxification (Synergism Bioassays; PBO)	[422]
D	Field	Malaysia	Taman Sungai Jelok, Malaysia	Institute for Medical Research, Malaysia Susceptible Laboratory Strain	Adult	WHO bioassay	KT_50_ (min)	1.01 to 6.85	N/A	[423]
D	Field	Malaysia	Selango, Malaysia	Vector Control Research Unit, a Malaysian susceptible strain	Adult	WHO bioassay	KT_50_ (min)	13.64	*Kdr* mutations (S989P. A1007G, V1016G) and metabolic detoxification (Synergism Bioassays; PBO)	[377]
D	Field	Indonesia	Palembang, Indonesia	Vector Control Research Unit, a Malaysian susceptible strain	Adult	WHO bioassay	LT_50_ (min)	1.05 to 12.75	N/A	[424]
P	Laboratory	-	Per-R (Sourced from Taiwan in 1990)	Unspecified	Larval	Larval Bioassay	LC_50_ (ng/mL)	394	*Kdr* mutations (V1016G and D1794Y)	[425]
P	Laboratory	-	F1 cross of SP females and SMK males	SMK	Adult	Topical Application	LD_50_ (ng/mosquito)	9.3	*Kdr* mutations (S989P, I1011M/V, L1014F, V1016G/I, F1534C, D1763Y), detoxification gene expression, and cuticle penetration	[179]
P	Laboratory	-	F1 cross of SMK females and SP males	SMK	Adult	Topical Application	LD_50_ (ng/mosquito)	8	*Kdr* mutations (S989P, I1011M/V, L1014F, V1016G/I, F1534C, D1763Y), detoxification gene expression, and cuticle penetration	[179]
P	Laboratory	-	Backcross of the F1 generation males (SP males and SMK females) and SMK females (BC1)	SMK	Adult	Topical Application	LD_50_ (ng/mosquito)	2.4	*Kdr* mutations (S989P, I1011M/V, L1014F, V1016G/I, F1534C, D1763Y), detoxification gene expression, and cuticle penetration	[179]
P	Laboratory	-	Backcross of the F1 generation females (SP females and SMK males) and SMK males (BC1)	SMK	Adult	Topical Application	LD_50_ (ng/mosquito)	3.3	*Kdr* mutations (S989P, I1011M/V, L1014F, V1016G/I, F1534C, D1763Y), detoxification gene expression, and cuticle penetration	[179]
P	Laboratory	-	Congenic strain from that expresses both the *cyp*-mediated genes and VGSC mutations S989P + V1016G. (CKR)	Rockefeller	Adult	Topical Application	LD_50_ (ng/mosquito)	112	*Kdr* (S989P, V1016G) and detoxification gene expression	[181]
P	Laboratory	-	Congenic strain from Singapore that expresses only the *cyp*-mediated genes (SP)	Rockefeller	Adult	Topical Application	LD_50_ (ng/mosquito)	356	Detoxification; gene expression	[181]
P	Laboratory	-	Congenic strain that expresses only the *cyp*-mediated genes (CR)	Rockefeller	Adult	Topical Application	LD_50_ (ng/mosquito)	6.17	Metabolic detoxification (Synergism Bioassays; PBO)	[339]
P	Laboratory	USA	Key West, FL, USA (Selected for Permethrin Resistance)	Collected from Key West, FL, USA	Adult	CDC bottle bioassay	LD_50_ (µg/bottle)	23.7	*Kdr* mutations (V1016I, F1534C) and detoxification gene expression	[426]
P	Laboratory	USA	Permethrin-selected Laboratory Strain	Lab Strain Originally collected in Koala Lumpur	Adult	WHO bioassay	LC_50_ (mg/L)	1.5	Enzymatic activity	[427]
P	Laboratory	Mexico	Acapulco, Guerrero, Mexico	New Orleans	Adult	Bottle bioassay	LC_50_ (µg/bottle)	1.19 to 38.59	*Kdr* mutations (V1016I, F1534C)	[404]
P	Laboratory	Puerto Rico	Pyrethroid-resistant Puerto Rican strain (PR)	Orlando	Adult	Topical Application	LD_50_ (µg/insect)	73.07	Detoxification; gene expression	[428]
P	Laboratory	Puerto Rico	PR after 20 generations of permethrin selection	Orlando	Larval	Larval Bioassay	LC_50_ (ppm)	6500	Metabolic detoxification (Synergism Bioassays; PBO, DEM, or DEF)	[205]
P	Laboratory	Brazil	MCNaeg-LIC	SMK	Adult	Topical Application	LD_50_ (ng/mosquito)	24	*Kdr* mutations (V253F, M374I, V410L, S723T, G923S, V1016I, F1534C)	[265]
P	Laboratory	Brazil	MCNaeg-C	SMK	Adult	Topical Application	LD_50_ (ng/mosquito)	42	*Kdr* mutations (V253F, M374I, V410L, S723T, G923S, V1016I, F1534C)	[265]
P	Laboratory	Brazil	Recife Colony-U: strain without insecticide exposure	New Orleans	Adult	WHO tube assays	LC_50_ (mg/m^2^)	2.41	*Kdr* mutations (V1016I, F1534C) and detoxification gene expression	[186]
P	Laboratory	Brazil	Recife Colony-R (Selected with temephos)	New Orleans	Adult	WHO tube assays	LC_50_ (mg/m^2^)	3.01	*Kdr* mutations (V1016I, F1534C) and detoxification gene expression	[186]
P	Laboratory	Brazil	Recife Colony-P (Selected with malathion)	New Orleans	Adult	WHO tube assays	LC_50_ (mg/m^2^)	11.09	*Kdr* mutations (V1016I, F1534C) and detoxification gene expression	[186]
P	Laboratory	Colombia	Acaci, Colombia	Rockefeller	Larval	Larval Bioassay	LC_50_ (ppm)	87.55	*Kdr* mutations (V410L, V1016I, F1534C) and enzymatic activity	[371]
P	Laboratory	Colombia	Acaci, Colombia	Rockefeller	Larval	Larval Bioassay	LC_50_ (ppm)	1608.3	*Kdr* mutations (V410L, V1016I, F1534C) and enzymatic activity	[371]
P	Laboratory	Taiwan	LYPR strain: Permethrin resistant 4th instars, formerly Lingya 1990R	Taiwanese local Susceptible strain; Originally Collected in 1987	Adult	WHO bioassay	LC_50_ (g/m^2^)	73	*Kdr* mutations (V1016G and D1794Y) and enzymatic activity	[429]
P	Laboratory	Singapore	SPS 0–10 (A pyrethroid-resistant population selected with permethrin for ten generations)	SMK	Adult	Topical Application	LD_50_ (ng/mosquito)	35 to 1650	*Kdr* mutations (S989P, I1011M/V, L1014F, V1016G/I, F1534C, D1763Y), detoxification gene expression, and cuticle penetration	[179]
P	Laboratory	Indonesia	Aceh, Indonesia	Bora-Bora	Adult	WHO bioassay	KT_50_ (min)	4.08 to 127	Metabolic detoxification (Synergism Bioassays; PBO or DEF)	[400]
P	Laboratory	French Polynesia	Paea, Tahiti, French Polynesia	Rockefeller	Larval	WHO larval bioassay	LC_50_ (mg/L)	1.8	N/A	[401]
P	Field	USA	Harris County, TX, USA	Orlando	Adult	Topical Application	LD_50_ (ng/mg of mosquito)	3.7 to 34.3	*Kdr* mutations (small argument for behavioral, but its more behavioral V1016I, F1534C) and detoxification gene expression	[430]
P	Field	USA	Anna Maria Island, FL, USA	Orlando	Adult	Topical Application	LD_50_ (ng/mg of mosquito)	6 to 56.8	*Kdr* mutations (V1016I, F1534C)	[431]
P	Field	USA	Montclair, California, USA	Navy Entomology Center of Excellence (Jacksonville, FL)	Adult	Bottle bioassay	LC_50_ (ppm)	1.13	N/A	[432]
P	Field	USA	St. Augustine, Florida, USA	Orlando	Larval	Larval Bioassay	LC_50_ (ppm)	25	Metabolic detoxification (Synergism Bioassays; PBO, DEM, or DEF)	[205]
P	Field	USA	St. Augustine, Florida, USA	Orlando	Adult	CDC bottle bioassay	LD_50_ ( ng/mg)	4.2–60.7	*Kdr* mutations (V1016I, F1534C) and enzymatic activity	[166]
P	Field	Mexico	Panuco, Veracruz, Mexico	New Orleans	Adult	Bottle bioassay	LC_50_ (µg/bottle)	1.95 to 33.23	N/A	[402]
P	Field	Mexico	San Luis Acatlán, Guerrero, Mexico	New Orleans	Adult	CDC bottle bioassay	LC_50_ (mg/L)	1.43 to 4.5	N/A	[403]
P	Field	Puerto Rico	Ponce, Puerto Rico	New Orleans	Adult	Bottle bioassay	LC_50_ (µg/bottle)	33 to 214	*Kdr* mutations (V1016I, F1534C)	[433]
P	Field	Colombian Caribbean Region	Puerto Colombia, Atlántico, Colombian Caribbean Region	Rockefeller	Adult	CDC bottle bioassay	KC_50_ (µg/bottle)	1.2 to 30.8	*Kdr* mutations (V1016I) and enzymatic activity	[408]
P	Field	Argentina	Salvador Mazza, Argentina	Rockefeller	Adult	WHO bioassay	LC_50_ (% a.i. /paper)	10.3	N/A	[434]
P	Field	Brazil	Belem, Brazil	Bora-Bora	Larval	Larval Bioassay	LC_50_ (ng/L)	4.2	*Kdr* mutations (G923V, L982W, I1011M, V1016G)	[269]
P	Field	Brazil	Boa Vista, Roraima State, Brazil	Rockefeller	Adult	WHO tube assays	KT_50_ (min)	2.5 to 5.9	*Kdr* mutations (V1016I, F1534C)	[435]
P	Field	Colombia	Bello, Colombia	Rockefeller	Larval	WHO larval bioassay	LC_50_ (ppm)	13.82 to 152.07	*Kdr* mutations (V410L, V1016I, F1534C) and enzymatic activity	[371]
P	Field	Peru	Iquitos, Perú	New Orleans	Adult	Bottle bioassay	LC_50_ (µg of a.i. / bottle)	2.1 to 10.2	*Kdr* mutations (V1016I) and detoxification gene expression	[201]
P	Field	West Africa	National Zoological Park, Abidjan, Côte d’Ivoire, West Africa	Bora-Bora	Adult	WHO bioassay	KT_50_ (min)	1.8 to 2.22	N/A	[410]
P	Field	West Africa	Adobo, Abidjan, Côte d’Ivoire, West Africa	Benin (Susceptible Strain)	Adult	WHO bioassay	KT_50_ (min)	1.62 to 2.03	*Kdr* mutations (V410L, V1016I, and F1534C)	[411]
P	Field	Pakistan	Faisalabad, Pakistan, Urban	Susceptible laboratory strain from Bahawalpu, Pakistan	Adult	WHO (Contact)	LC_50_ (ppm)	1.41 to 19.74	Enzymatic activity	[412]
P	Field	Pakistan	Faisalabad, Punjab, Pakistan	Susceptible Lab Strain Collected Indigenously	Adult	CDC bottle bioassay	KC_50_ (µg/mL)	5.84 to 38.23	Metabolic detoxification (Synergism Bioassays; PBO or DEF)	[415]
P	Field	India	Jaipur, Rajasthan, India	Unspecified	Adult	CDC bottle bioassay	LC_50_ (ppm)	2	N/A	[436]
P	Field	India	Dharmapuri, India	Susceptible strain from the National Centre for Disease Control (NCDC), Mettupalayam	Adult	WHO bioassay	LT_50_ (min)	5.1 to 6	*Kdr* mutations (F1534C) and metabolic detoxification—Synergism Bioassays (PBO or DEF) and Enzymatic activity	[437]
P	Field	Sri Lanka	Colombo, Sri Lanka	New Orleans	Adult	Bottle bioassay	LC_50_ (µg/bottle)	2.6 to 12.9	*Kdr* mutations (S989P, V1016G, F1534C) and enzymatic activity	[438]
P	Field	Malaysia	Kota Bharu, Kelantan, Malaysia	Vector Control Research Unit, a Malaysian susceptible strain	Adult	WHO bioassay	KT_50_ (min)	115.88	*Kdr* mutations (V1016G, F1534C)	[439]
P	Field	Malaysia	Kuala Lumpur, Malaysia	Insecticide susceptible strain from Selango	Adult	WHO bioassay	LT_50_ (min)	1.48 to 1.88	*Kdr* mutations (S989P, V1016G, F1534C)	[440]
P	Field	Malaysia	Gombak, Selangor, Malaysia	Bora-Bora	Adult	CDC bottle bioassay	KT_50_ (min)	0.84 to 31.75	*Kdr* mutations (S989P, V1016G, F1534C) and metabolic detoxification—Synergism Bioassays (PBO, EA, or DEF) and Enzymatic activity	[331]
P	Field	Malaysia	Penang Island, Malaysia	Institute for Medical Research, Malaysia Susceptible Laboratory Strain	Adult	WHO bioassay	KT_50_ (min)	12.37	Metabolic detoxification (Synergism Bioassays; PBO)	[422]
P	Field	Malaysia	Ridzuan Condominium, Selangor, Malaysia	Institute for Medical Research, Malaysia Susceptible Strain	Adult	WHO bioassay	KD_50_ (min)	0.51 to 593.10	N/A	[441]
P	Field	Malaysia	Dengue hotspot, Penang Island, Malaysia	Vector Control Research Unit, a Malaysian susceptible strain	Adult	WHO bioassay	KT_50_ (min)	4.0 to 7.0	Differential gene expression	[442]
P	Field	Malaysia	Taman Sungai Jelok, Malaysia	Institute for Medical Research, Malaysia Susceptible Laboratory Strain	Adult	WHO bioassay	KT_50_ (min)	2.66 to 12.7	N/A	[423]
P	Field	Malaysia	Taman Melati Zone of Kuala Lumpur, Malaysia	Lab Strain Originally collected in Koala Lumpur	Adult	WHO bioassay	LC_50_ (mg/L)	4.28 to 4.47	Enzymatic activity	[427]
P	Field	Malaysia	Selango, Malaysia	Vector Control Research Unit, a Malaysian susceptible strain	Adult	WHO bioassay	KT_50_ (min)	58.93	*Kdr* mutations (S989P. A1007G, V1016G) and metabolic detoxification (Synergism Bioassays; PBO)	[377]
P	Field	Singapore	Ang Mo Kio, Singapore	Bora-Bora	Adult	WHO bioassay	KT_50_ (min)	27.58 to 64.96	Detoxification enzymes	[418]
P	Field	Singapore	Ang Mo Kio, Singapore	Bora-Bora	Adult	WHO bioassay	LC_50_ (mg/L)	29 to 47	Metabolic detoxification—Synergism Bioassays (PBO, DEF, TPP) and Enzymatic activity	[443]
P	Field	Taiwan	Guanmiao District strain, from Tainan City, Taiwan	NS (Taiwanese local susceptible strain;)	Adult	WHO bioassay	LC_50_ (g/m^2^)	10.0 to 57.0	*Kdr* mutations (V1016G and D1794Y) and enzymatic activity	[429]
P	Field	Taiwan	Annan, Taiwan	Gainesville Strain (From USDA)	Adult	WHO bioassay	KT_50_ (min)	3.35 to 184.07	Behavioral responses	[419]
P	Field	Thailand	Amnat Charoen, Thailand	Bora-Bora	Adult	WHO tube assays	LT_50_ (min)	10.16 to 15.13	Enzymatic activity	[420]
P	Field	Vietnam	Long Hoa, Vietnam	Bora-Bora	Larval	Larval Bioassay	LC_50_ (ng/L)	77.5	*Kdr* mutations (G923V, L982W, I1011M, V1016G)	[269]
P	Field	Indonesia	Palembang, Indonesia	Vector Control Research Unit, a Malaysian susceptible strain	Adult	WHO bioassay	LT_50_ (min)	4.9 to 45.7	N/A	[424]
P	Field	Indonesia	Semarang, Indonesia	Bora-Bora	Larval	Larval Bioassay	LC_50_ (ng/L)	59.2	*Kdr* mutations (G923V, L982W, I1011M, V1016G)	[269]

*D* Deltamethrin, *P* Permethrin; RR* Resistance ratio obtained from different references calculated by dividing the toxicological metric [Lethal Dose (LD_50_), Lethal Concentration (LC_50_), Lethal Time (LT_50_), Knockdown Dose (KD_50_), Knockdown Concentration (KC_50_), Knockdown Time (KT_50_)] of the different populations by that of the susceptible reference strain used

When discussing laboratory-maintained strains, it is crucial to distinguish between susceptible strains, which are typically unexposed to pesticides and serve as controls, and resistant strains, which have undergone deliberate selection pressure from pesticides. Among laboratory-maintained strains, resistant strains subjected to prolonged selection pressures exhibited the most extreme levels of resistance. For permethrin, the highest RR (~6500) was recorded in a Puerto Rican strain following 20 generations of selection, where metabolic detoxification played a dominant role [205]. Field populations, although displaying lower RRs overall, still reached high resistance levels, with a maximum RR of ~593 observed in Malaysia, which had high frequencies of *kdr* mutations and metabolic resistance.

Deltamethrin resistance followed a similar trend, with exceptionally high RR values in laboratory-selected strains, including a peak of 17,000 in a Puerto Rican strain [205]. Among field populations, the highest deltamethrin RR ( ~ 249) was recorded in Malaysia, where both metabolic detoxification and *kdr* mutations were identified as contributing factors for the resistance [331]. While pyrethroid-selected laboratory strains can exhibit extreme resistance, field-collected populations frequently displayed high resistance levels (RR ≈ 10–250), reflecting ongoing selection pressure in natural environments.

Overall, there appears to be significant variability in pyrethroid resistance across different regions, with populations from the Americas, Asia, and Africa exhibiting diverse resistance mechanisms, most commonly metabolic detoxification and *kdr* mutations. However, most studies tend to focus on a single resistance mechanism, which may not capture the complexity of resistance in these populations or account for the interplay between multiple mechanisms [332]. Several countries, particularly in the Americas and parts of Asia, are well represented, with repeated entries from Mexico, Cuba, Brazil, and Thailand. However, the overall distribution is uneven, with limited contributions from many regions. Notably, Africa is sparsely represented despite its high burden of mosquito-borne diseases, and several countries across Latin America and Asia appear only once in the dataset. This underrepresentation underscores the need for broader geographic coverage and more inclusive surveillance to support global strategies in resistance management. Furthermore, the roles of cuticular alterations and behavioral adaptations in contributing to overall resistance remain relatively understudied and are often overlooked in resistance assessments [215]. Persistent selection of laboratory-resistant strains tend to exhibit the highest levels of resistance, and similarly, high RR values in field populations indicate active resistance evolution under continued pyrethroid exposure. These findings emphasize the urgent need for sustained resistance monitoring and integrated vector management strategies to curb the progression of pyrethroid resistance in *Ae. aegypti* populations worldwide.

### A comprehensive approach of assessing resistance

The practical application of bioassay pyrethroid-efficacy findings to ongoing control measures is critical for ensuring the effectiveness of pyrethroid-based interventions, as it allows for timely adoption of IVM approaches when resistance emerges. Resistance thresholds derived from bioassay results guide decisions on insecticide use, helping public health officials determine when to rotate to alternative chemicals or employ synergists Like piperonyl butoxide to counteract CYP 450s metabolic resistance [122]. However, operational constraints such as limited budgets and inconsistent application methods can impact the success of these interventions [20]. Addressing these challenges requires a comprehensive approach that combines bioassay findings with real-world considerations, such as the availability of resources and community acceptance of chemical interventions. Moreover, control measures must adapt to local resistance patterns.

Combining field and laboratory methods provides a comprehensive understanding of insecticide resistance dynamics. Early detection allows for timely adjustments in control strategies, such as rotating insecticide classes, using synergists, deploying alternative control methods, and implementing resistance management plans [333].

In the context of insecticide resistance management, baseline susceptibility levels, screening for common resistant genotypes and potential metabolic detoxification pathways, while tracking historical insecticide usage should be considered even when resistance is not detected. Identifying locations with predominantly susceptible populations is important, as the movement and interbreeding of individuals from these susceptible populations into the broader pest population can act as a critical mechanism for reducing the prevalence of emerging resistance alleles [260, 334]. Additionally, populations can undergo susceptibility reversion after prolonged periods of nonselection [335]. While such populations may be phenotypically susceptible, a slight heterozygous advantage under selective pressure can lead to a resurgence of resistant alleles and renewed resistance development. This issue is further complicated by some resistance genes exhibiting cross-resistance to various pesticides, while others do not [336–341]. Therefore, it is crucial to implement continuous surveillance to prevent the development of resistance.

#### Current knowledge for relating insecticide resistance and field efficacy for *Ae. aegypti*

Despite the growing understanding of insecticide resistance mechanisms and their significance for *Ae. aegypti* control*,* significant gaps remain in the current literature regarding field efficacy and the correlation of resistance bioassay results with practical control measures [342]. While other reports considered various variables, none incorporated bioassay tests and genotypic analyses correlated with survival. For instance, in *Ae. aegypti* from Mexico, mosquitoes with high frequencies of the 1534C And 1016I *kdr* mutations were more likely to survive indoor residual spraying of deltamethrin, but the amount of pesticide exposure was unquantified [343]. In São Paulo, Brazil, *Ae. aegypti* characterized as resistant in laboratory assays were subsequently confirmed as resistant in sentinel cages exposed to ULV treatment of cypermethrin in field efficacy tests, though the resistance mechanism was not investigated [344]. Indeed, many studies include both bioassays and *kdr* genotype analyses for *Ae. aegypti*, but their relationship cannot be fully assessed because the mosquitoes genotyped are either distinct from those used in bioassays or were analyzed in pooled samples, preventing direct genotype–phenotype correlation [182, 186, 345, 346]. A comprehensive study by Lee et al. [347] determined the field resistance ratio of field-collected *Culex quinquefasciatus* mosquitoes to Permanone^®^ (a commercial formulation; a.i., Permethrin 33% with synergist PBO 66%), comparing it to a pyrethroid-susceptible strain. This resistance ratio was consistent with those determined in laboratory vial assays with the same cohort of mosquitoes assayed in the field. This study is currently one of the few that directly links insecticide resistance ratios from laboratory bioassays with the effectiveness of operational control measures and pesticide deposited in the field, that is, with field resistance ratios [342].

**Fig.4:**
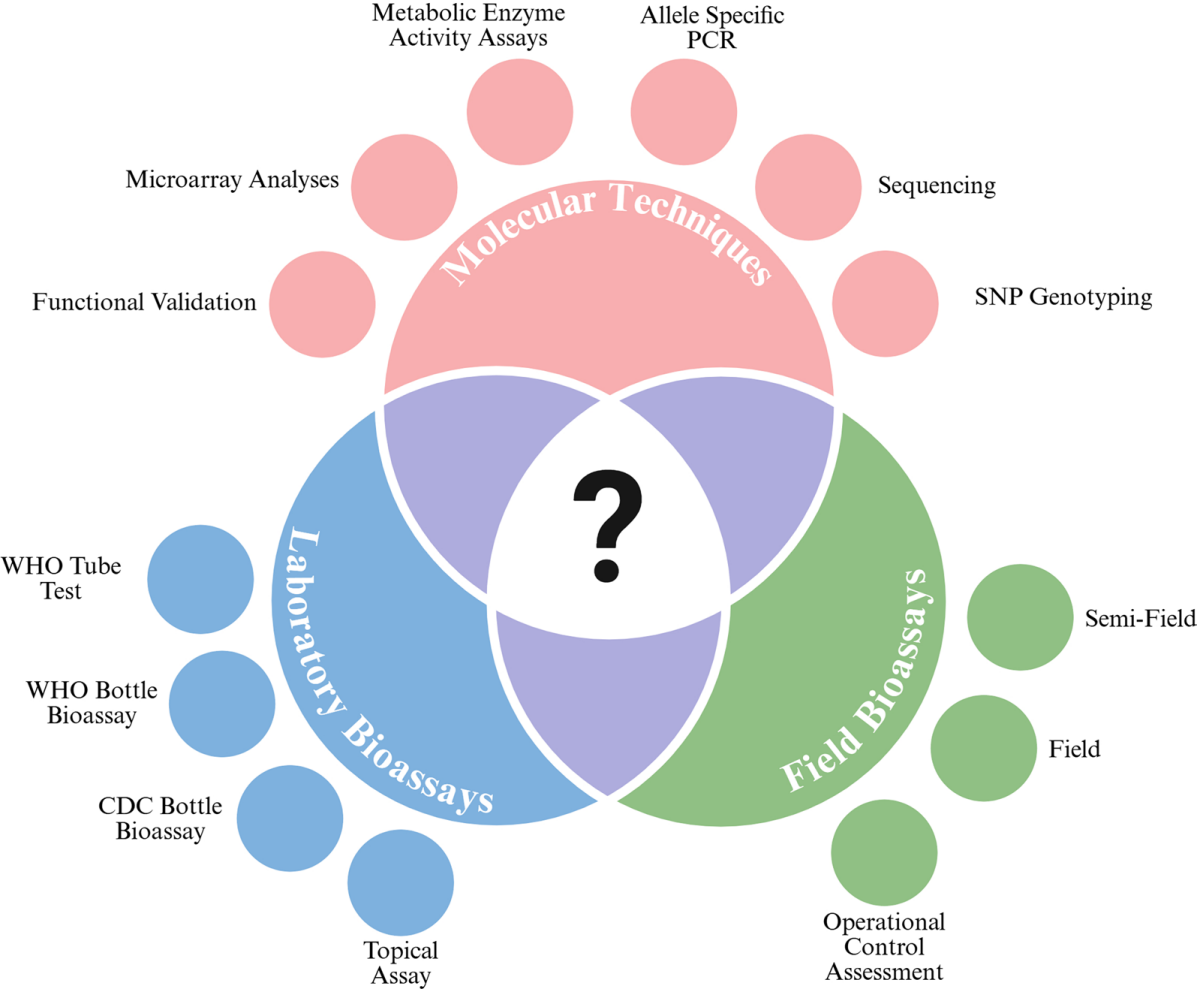
Venn diagram illustrating the different methodologies used to detect insecticide resistance in *Ae. aegypti*. There is currently no study that incorporates all three methodologies in *Ae. aegypti* to determine how resistance influences pyrethroid field efficacy, represented by the question mark in the center. This demonstrates the need for comprehensive studies that integrate all three methodologies to assess the impact of resistance on vector control

In Harris County, TX, USA, logistic regression models identified that variations in the amount of pesticide delivered in the field from a handheld fogger can differentially influence the mortality rates of pyrethroid-susceptible and field-collected mosquitoes in the field [171]. It was later determined that females of *Ae. aegypti* that possess *kdr* L allele(s) at the 410-site of the VGSC had increased Survivorship at 15.34 And 22.86 m distances from the spray source when treated with Permanone, while most died at 7.62 m [170]. These distances are relevant to the application parameters of the London Fog Colt-4 handheld sprayer used, which has An assumed application swath of 38.1 m [348]. The manufacturer’s manual recommends moving downwind approximately 15.34–22.86 m on each Successive pass if the area to be treated is over 38.1 m, indicating that the observed survivorship distances fall within the expected range of the sprayer’s operational effectiveness. Gas chromatography-mass spectrometry (GC–MS) identified and estimated the amount of permethrin + PBO delivered in the field at each post in a field cage test that exposed *Ae. aegypti* to Permanone applied with a ULV handheld fogger [169]. By quantifying the amount of pesticide, estimating control efficacy, and genotyping individual mosquitoes, this study linked the survival probabilities of mosquitoes with different *kdr* genotypes, and the amount of pesticide they received in the field. The various methodologies used to investigate insecticide resistance, encompassing laboratory bioassays, field bioassays, and molecular techniques, are summarized in a Venn diagram in Fig. 4. However, the relationship between phenotypic results in the field and laboratory assays, when considering genotypic resistance, remains unknown in *Ae. aegypti*. Future investigation into this intersecting relationship is crucial to bridge this critical gap.

### Conclusions and tactics toward improved IVM

This review highlights the critical need for more effective IVM strategies, especially for managing disease outbreaks in regions where *Ae. aegypti* poses a significant public health threat. While pyrethroid resistance remains a central focus, alternative control strategies, including other chemical modes of action, biological control, and genetic approaches, are being explored in parallel to complement chemical interventions [349, 350]. These strategies should be positioned as complementary to the last resort, pyrethroid-based interventions rather than replacements, helping to reduce reliance on chemical controls and demonstrating the value of combining immediate chemical interventions with long-term sustainable solutions.

While the information presented here has contributed significantly to our understanding of genotypic resistance and its impact on the control efficacy of pyrethroids against females of *Ae. aegypti*, there remain critical areas that require further exploration. It is important to investigate other underexplored resistance mechanisms, such as cuticle-based resistance and behavioral modification. There are also novel mechanisms that may contribute to resistance alongside those previously established [351]. Bioassays will remain crucial for assessing insecticide resistance in mosquito populations, providing baseline data to inform control measures. To improve predictive power, bioassays should incorporate field-relevant variables, including exposure time and environmental conditions, while being integrated with molecular diagnostics to confirm resistance mechanisms. Ultimately, further research is needed to understand the impact of insecticide resistance in vector control strategies [142].

#### Recommendations for effective and adaptive vector control amidst widespread pyrethroid resistance

The global emergence of pyrethroid resistance in *Aedes aegypti* significantly compromises the effectiveness of vector control programs [144, 195, 228]. In the USA, a 2017 report from the National Association of County and City Health Officials (NACCHO) on the mosquito Surveillance And control assessment of 1083 vector control organizations that responded to the Survey indicated that 84% of them needed improvement. This was based on the self-evaluation of five “core competencies” that were: (1) routine mosquito surveillance through standardize trapping and species identification; (2) treatment decisions using surveillance data; (3) Larviciding, adulticiding or both; (4) routine vector control activities; (5) pesticide resistance testing [352]. Of the vector control programs ranked as “needs improvement” 98% lack the capability to perform pesticide resistance testing.

Addressing this critical challenge requires a strategic and multifaceted approach grounded in the principles of IVM (discussed in the “Integrated vector management practices” section) [353–355]. Critically, these strategies must be adaptable, as not all countries have access to the same financial, technological, or human resources. Public willingness to fund vector control is a key factor in the financial feasibility of local programs [356, 357]. A cornerstone of this adaptive strategy is robust, data-driven surveillance and insecticide resistance management (IRM). Routine monitoring of pyrethroid susceptibility in local vector populations across geographically defined operational areas is essential for making evidence-based decisions, i.e. treatments based on surveillance [142, 358]. This includes mapping the spatial distribution of resistance genotypes and identifying geographic hotspots where these genotypes associated with higher levels of resistance are most frequent. Under pyrethroid selection pressure, these alleles can become fixed and spread rapidly, impairing the long-term effectiveness of pyrethroid control. Ideally, the spatial distribution of frequency and levels of resistance should be published on public websites to provide homeowners and private applicators with actionable guidance for preventing resistance spread. It would also be important to designate a few operational areas for insecticide resistance surveillance over several years, the number of these areas depending on the human population size, resources, and historical detection of viruses or similar factors. The operational areas for this long-term surveillance could be those with the lowest and highest levels of resistance to evaluate the effectiveness of resistance management interventions through time.

The current over-reliance on chemical adulticides is unsustainable. Programs should, therefore, integrate environmental management of larval habitats as it targets mosquitoes in contained aquatic environments before they can emerge and transmit disease [359]. The use of bacterial larvicides, such as *Bti*, and insect-growth regulators remains a highly effective strategy where local susceptibility persists [92, 360, 361]. Furthermore, empowering communities to identify and eliminate mosquito breeding sites is a cost-effective and sustainable strategy that reduces the need for chemical interventions and builds public trust [362]. Expanding the use of non-chemical methods, such as lethal ovitraps and adult traps, can further help reduce mosquito populations without contributing to insecticide resistance [363]. The integration of novel vector control technologies, including Wolbachia-infected mosquito releases, sterile insect techniques (SIT), and gene drive, offers promising avenues for overcoming pyrethroid resistance by reducing reliance on chemical insecticides and suppressing resistant mosquito populations [364].

When surveillance programs detect pyrethroid resistance, programs should quickly reorient to incorporate different modes of action or mixtures to reduce selection pressure. Effective IRM protocols incorporate the systematic rotation of insecticides from different IRAC classes [228, 365, 366]. For instance, organophosphates or microbial larvicides can be used in rotation or combination with pyrethroids deployed. In areas where resistance hot-spots are confirmed, adulticide campaigns should prioritize resistance-breaking mixtures, such as a recently approved triple-action formulation combining a pyrethroid with abamectin and PBO (e.g., ReMoa Tri), which has demonstrated efficacy against pyrethroid-resistant *Aedes* populations [95]. Conversely, operational areas identified with the lowest frequency or levels of resistance should be preserved through IVM by minimizing unnecessary adulticide use and maintaining susceptibility by planned rotations or by withholding pyrethroids unless epidemiologically justified [142].

Effective strategic management of available insecticides is crucial for mitigating resistance. However, a global review of insecticide use found that many national programs were slow to withdraw pyrethroids even after laboratory confirmation of resistance, and the proactive use of rotations or mixtures was generally weak [228]. This disconnection between knowledge and action not only wastes resources on ineffective interventions but also exerts continuous selection pressure, which in turn accelerates the complete loss of pyrethroids as a viable public health tool. Closing this gap between evidence and implementation is critical for global public health.

The challenge of pyrethroid resistance demands a comprehensive and adaptive vector control strategy. By moving away from a sole reliance on pyrethroids, programs can build a resilient and effective response. This integrated approach combining data-driven surveillance, strategic insecticide deployment, strong community education and operational responsiveness is essential to combat mosquito-borne diseases and preserve the efficacy of our limited chemical tools for the future.

## Data Availability

Data supporting the main conclusions of this study are included in the manuscript.
